# *OsCIPK2* mediated rice root microorganisms and metabolites to improve plant nitrogen uptake

**DOI:** 10.1186/s12870-024-04982-0

**Published:** 2024-04-16

**Authors:** Mengying Chen, Shizhong Feng, He Lv, Zewen Wang, Yuhang Zeng, Caihong Shao, Wenxiong Lin, Zhixing Zhang

**Affiliations:** 1 College of JunCao Science and Ecology, Fujian Agricultural and Forestry University, Fuzhou, Fujian 350002 China; 2 Key Laboratory of Crop Ecology and Molecular Physiology, Fujian Agriculture and Forestry University, Fuzhou, Fujian 350002 China; 3 Fujian Provincial Key Laboratory of Agroecological Processing and Safety Monitoring, Fujian Agriculture and Forestry University, Fuzhou, 350002 China; 4 Soil and Fertilizer & Resources and Environment Institute, Jiangxi Academy of Agricultural Sciences, Nanchang, 330200 China

**Keywords:** *OsCIPK2*, Nitrogen uptake, Rhizosphere soil, Root microbiome, Metabolites, Synthetic microbial communities

## Abstract

Crop roots are colonized by large numbers of microorganisms, collectively known as the root-microbiome, which modulate plant growth, development and contribute to elemental nutrient uptake. In conditions of nitrogen limitation, the over-expressed *Calcineurin B-like interacting protein kinase 2* (*OsCIPK2*) gene with root-specific promoter (RC) has been shown to enhance growth and nitrogen uptake in rice. Analysis of root-associated bacteria through high-throughput sequencing revealed that *OsCIPK2* has a significant impact on the diversity of the root microbial community under low nitrogen stress. The quantification of *nifH* gene expression demonstrated a significant enhancement in nitrogen-fixing capabilities in the roots of RC transgenetic rice. Synthetic microbial communities (SynCom) consisting of six nitrogen-fixing bacterial strains were observed to be enriched in the roots of RC, leading to a substantial improvement in rice growth and nitrogen uptake in nitrogen-deficient soils. Forty and twenty-three metabolites exhibiting differential abundance were identified in the roots and rhizosphere soils of RC transgenic rice compared to wild-type (WT) rice. These findings suggest that *OSCIPK2* plays a role in restructuring the microbial community in the roots through the regulation of metabolite synthesis and secretion. Further experiments involving the exogenous addition of citric acid revealed that an optimal concentration of this compound facilitated the growth of nitrogen-fixing bacteria and substantially augmented their population in the soil, highlighting the importance of citric acid in promoting nitrogen fixation under conditions of low nitrogen availability. These findings suggest that *OsCIPK2* plays a role in enhancing nitrogen uptake by rice plants from the soil by influencing the assembly of root microbial communities, thereby offering valuable insights for enhancing nitrogen utilization in rice cultivation.

**Supplementary Information:**

The online version contains supplementary material available at 10.1186/s12870-024-04982-0.

## Introduction

To ensure food security for the growing population, agricultural systems must produce crops that are environmentally friendly [1]. Soil rhizomicrobiomes promote plant growth and health, making them important to dealing with this challenge [2–4]. Plant-associated microbiota colonize diverse tissue types and root surfaces to facilitate access to soil nutrients [5]. The structure of microbial communities is significantly influenced by both plant genotypes and environmental factors [6, 7]. In response to biotic or abiotic stress, plants secrete chemical factors to help them adapt to these stresses by recruiting beneficial microorganisms, which is known as the “cry for help” strategy [5, 8]. Crop roots are colonized by large numbers of microorganisms, collectively known as the root-microbiome, which modulate plant growth as well as development and contribute to plant fitness under diverse environmental conditions [9, 10]. Roots also secret organic acids, amino acids, and sugars, which provide microorganisms in the rhizosphere with rich nutrients, enabling microbiota to migrate into the rhizosphere and roots [11]. Apart from nutrient uptake, the root-microbiome promotes plant growth and development by modulating plant hormone homeostasis, improving resilience to abiotic stresses, or protecting the host from pathogens [12, 13].

The growth and development of crops heavily rely on availability of nutrients that the root system can access, implying that crops have to overcome many challenges in extracting nutrients for cellular functions, and any lack of nutrients may decrease productivity [14, 15]. To enhance their defense abilities against nutrient deprivation, crop species recruit microbes from soil by regulating their nutrient uptake genes [16, 17]. For example, the maize *rum1* gene is an important checkpoint for auxin-mediated initiation of lateral and seminal roots, which regulates auxin transport in primary roots as well as auxin perception in primary root pericytes and influences lateral root development [18]. Mutant *rum1* lacks lateral roots, which reduces water and nutrient acquisition during early development. Compared with wild-type maize, bacterial diversity in the mutant *rum1* rhizosphere was found to be significantly reduced during root development [19]. These results show that the rhizosphere’s microbial composition may be influenced by root development-related genes through a number of signaling substances that control lateral root’ length and number.

China accounts for about 20% of the world’s rice planting area, and consumes nearly 40% of the total nitrogen fertilizer used globally [20]. In the last 30 years, nitrogenous fertilizer consumption in China has increased 3.28-fold; however, nitrogen use efficiency (NUE) is about 30–35%, which is less than the global average value (40–60%) [21, 22]. Plants take up nitrogen through their roots as nitrates (NO_3_^−^) and ammonia (NH_4_^+^), which they actively use in their metabolic processes. Various NUE-related genes have been identified, providing valuable information for screening or molecular breeding of high-NUE rice cultivars, such as *Nitrate Transporter 1(NRT1*), *DENSE AND ERECT PANICLE 1(DEP1)*, *NUE-related transcription factor 42* (*NAC42)* and *Glutamine synthetase1.1 (GS1.1)* [23–25]. Recently, more and more research showed that Calcineurin B-like proteins (CBLs) and CBL-interacting proteinkinases (CIPKs) play an important role in nitrogen uptake and root development. For example, the *CmCIPK23*, a CIPK from Chrysanthemum, regulates *CmTGA1* and activates nitrogen uptake during root development [26]. In plants, CBL and CIPK proteins form one of the important Ca^2+^ decoding complexes to decipher Ca^2+^ signals elicited by environmental challenges, such as nitrogen-limiting stress [27, 28]. When *Arabidopsis* plants are subjected to low NO_3_^-^ concentrations, CBL9-CIPK23 complexes can phosphorylate the Thr101 site of CHL1, the earliest identified nitrate transporter in plants, enhancing NO_3_^-^ uptake by plants [29]. Meanwhile, CIPK23 regulates NH_4_^+^ uptake by phospho-regulating AMT1, a high-affinity NH_4_^+^ transporter [30]. Moreover, *CIPK8* positively regulates early nitrate signaling in *Arabidopsis* [31]. In a previous study under nitrogen-limiting conditions, we also found that *OSCIPK2*-overexpressing transgenic rice plants with root specific promoter (RC) increased rice yields by 35% and nitrogen uptake by 38.51%, compared to wild type (WT) rice [32].

Recent research has demonstrated that plant NUE-related genes play a crucial role in modulating nitrogen uptake and transport by influencing the root microbial community. For instance, *NRT1.1B* facilitates the colonization of rice roots by nitrogen cycle bacteria and promotes the conversion of organic nitrogen [33]. Endophytes microbes (e.g. root microbiomes) are ubiquitous in plant species and may originate from the soil, colonizing plants internally after successful interaction in the rhizosphere. These endophytes provide various benefits to plants, including increased nutrient uptake by roots, enhanced defense mechanisms, stress alleviation and modulation of plant development [34]. Recently study showed that Ca^2+^ signals play a key role in beneficial plant-microbe associations, such as in rhizobia-legume symbiosis [35] and arbuscular mycorrhizal fungi (AMF) growth [36]. In a study by Bao et al., it was found that the use of a calcium-based fertilizer led to a significant increase in the colonization rate of maize roots by AMF, with rates improving by up to 40% [37]. Given the significance of the CIPK family in mediating Ca^2+^ signaling, we propose that the overexpression of *OSCIPK2* in rice roots may enhance root colonization by specific microbial species, thereby promoting nitrogen uptake under nitrogen-deficient conditions.

This study utilized 16 S rRNA high-throughput sequencing to investigate the impact of genetic variations on the composition of root-associated microbial communities in nitrogen-limited soil conditions. The predominant bacterial strains enriched in RC roots were isolated and their functions validated using the SynCom system. Subsequently, metabolomics analyses of both root and rhizosphere soils were performed to identify the key metabolites influencing the formation of root-associated microbiomes. Our findings are conducive for reducing the amount of applied nitrogen fertilizers and increasing rice productivity as well as nutrient uptake.

## Materials and methods

The present study uses *OSCIPK2*-overexpressing transgenic rice plants to investigate how plant genes and root-associated microbes affect nitrogen uptake. Field and pot experiments were conducted under different nitrogen levels to analyze genetic variations in root microbiomes.

### Plant materials and growth conditions

The experiment was performed in the experimental farm (26°08′ N,119°28′ E) of Fujian Agriculture and Forestry University, Fuzhou, China during the rice growing season of 2018–2019. The *OSCIPK*2-overexpressing transgenic rice plant with root specific promoter (RC), which exhibits high nitrogen uptake efficiency [32], and its corresponding wild type (WT) were used as experimental materials. The WT cultivar Kitaake were obtained from the International Rice Research Institute, Los Banos, Philippines. At room temperature, rice seeds were soaked in water for 24 h at room temperature and germinated under moist conditions at 37℃ for 48 h. Germinated seeds were grown in paddy fields. After the 21-day germination period, seedlings were transplanted in a filed at a spacing of 0.15 × 0.15 m and one seedling per hill. Four nitrogen fertilizer levels were set (0 kg·ha^− 1^, 75 kg·ha^− 1^, 150 kg·ha^− 1^ and 225 kg·ha^− 1^), using urea as the nitrogen fertilizer. Phosphorus was applied as the base fertilizer and potassium for top dressing at rates of 112.5 kg ha^− 1^ (P_2_O_5_) and 180 kg ha^− 1^ (K_2_O), respectively. Soil textures included sandy loam, tillage layer with 2.33 g·kg^− 1^ total nitrogen, 196.2 mg·kg^− 1^ available nitrogen; 1.12 g·kg^− 1^ total phosphorus; 139.5 mg·kg^− 1^ available potassium; 1.05 g·kg^− 1^ total potassium; 135.09 mg·kg^− 1^ available phosphorus and 30.64 g·kg^− 1^ soil organic matter. In the soil pot experiment, five seedlings of RC and WT were transplanted in plastic buckets containing 12 kg soil. The RC and WT were separated by vertical plastic sheets in plastic buckets. The soils used in the pot experiment were consistent with those of the field experiment. No nitrogen fertilizer (0 kg·ha^− 1^) was applied during the whole growth season in the pot experiment. The amount of phosphorus and potassium fertilizer were converted according to the field experiment. The soil pot experiments were performed in a greenhouse at natural temperature and light from May 2019 to September 2019. Twenty pots were used in this experiment.

### Determination of physiological parameters and grain yields

To determine nitrogen levels and grain yields in the field experiment, 10 rice plants were dried to a constant weight and sampled three times for each sample. Yield components, including the number of spikelets per panicle, effective panicles, seed setting rate and 1000 grain weight were also determined. Rice samples in the soil pot were collected during heading and maturing stages. At the heading stage, total nitrogen levels, shoot lengths, total dry mass and chlorophyll levels of leaf were measured. The semi-micro Kjeldahl method was used to determine nitrogen and protein levels. To measure the chlorophyll levels, readings of flag leaves were measured using SPAD (soil–plant analyses development) 502 chlorophyll meter at 11:00–12:00 a.m.

### DNA extraction, PCR amplification and sequencing

Rice roots from the pot were obtained for bacterial 16sRNA gene profiling. Root sampling was performed at the heading stage and washed until there were no visible soil particles. Next, 10 cm long roots from the ground were sliced into 2 mm sections and placed in a 2-ml tube. Three replicates were performed for each sample. Total DNA extraction from root bacteria was performed using the BioFast soil Genomic DNA Extraction kit (BioFlux, Hangzhou, China), as instructed by the manufacturer. Total DNA from root bacteria were detected by 1% agarose electrophoresis and their concentrations determined by the NanoDrop 2000 nucleic acid analyzer (Thermo Scientific, USA). The V5-V7 region of bacterial 16 S ribosomal RNA gene was amplified using 799 F (AACMGGATTAGATACCCKG) and 1193R (ACGTCATCCCCACCTTCC) primers. The PCR assay was performed using Trans Start Fastpfu DNA Polymerase (TransGen Biotech, China). After amplification, PCR products were identified by electrophoresis on 2% agarose gel and recovered using the AxyPrepDNA Gel Recovery Kit (Axygen Bioscience, China) after elution using Tris-HCl. The library was sequenced on a HiSeq 2500 platform (Illumina, San Diego, CA, USA).

### Processing of high-throughput sequencing data

Raw data were first screened after which sequences were removed from consideration if they were shorter than 230 bp, had a low quality score (≤ 20), contained ambiguous bases or did not exactly match primer sequences as well as barcode tags, and separated using sample-specific barcode sequences. Reads were clustered into operational taxonomic units (OTUs) using the Vsearch’s Uparse algorithm (v2.7.1) at a similarity level of 97% [38]. All sequences were analyzed against the GenBank non-redundant nucleotide (nt) database using the BLAST tool.

The OTU information was used to generate rarefaction curves and to calculate the richness as well as diversity indices. α- andβ- diversities were analyzed using Mothur (v1.31.2) [39] and QIIME (v1.8.0) [40]. Heatmaps were generated using Mothur (version 1.31.2) to compare bacterial community membership and structures among samples [41]. To assess the similarity between RC and WT, clustering analyses and PCA were performed by R (v3.6.0) based on OTU information from RC and WT [42].

### Quantitative PCR analysis

A quantitative real-time PCR (qPCR) assay was performed using the Mastercycler ep realplex (Eppendorf, Germany) to determine the abundance of *nirH* genes and the specifically root-associated bacterial strains. The *nirH* was quantified with the primers (forward: 5’-CCTACGGGAGGCAGCAG-3’; reverse: 5’- ATTACCGCGGCTGCTGGCA-3’). The specifically root-associated bacterial strains were quantified using the primers shown in Table S1. The abundances of *nifH* and specifically root-associated bacterial strains in roots were quantified relative to a standard curve for plasmids containing the target gene or bacterial strains sequence inserts. qPCR was performed in a 15 µl reaction mixture containing 7.5 µl 2×q-PCR mastermix (DBI Bioscience, Germany), 0.6 µl of each primer (10 µ M) and 1 µl of template DNA (20 ng of total root or soil DNA or a serial dilution of plasmid DNA for standard curves). Four independent qPCR assays were performed for each sample.

### Determination of the effects of root-associated bacteria on plant growth

SynCom were designed to evaluate the effects of specific bacteria enrichment in RC roots. Six root-associated bacterial strains (*Phenylobacterium* sp., *Rhizobium* sp., *Pleomorphomonas* sp., *Devosia* sp., *Sphingomonas* sp. and *Azospirillum* sp.), were purchased from the Agricultural Culture Collection of China (ACCC, http://www.accc.org.cn/), and used to design a SynCom. The signal bacterial strain was cultivated in 50 ml tubes TSB medium at 28 °C and grown to OD 600 nm = 1. Bacterial cells were collected by centrifugation and resuspended in deionized water. The SynCom was created by inoculating six prepared bacterial suspensions in equal volumes, followed by adjusting them to an OD600 of 0.5 using deionized water. In the pot experiments, 40 ml of the SynComs suspensions was inoculated into the 250 g of native or sterile soil. The seeds of WT or RC transgenics rice were disinfected using 30% H_2_O_2_ for 30 min, rinsed 3–5 times using deionized water and placed in a dark incubator at 30 °C for 48 h to germinate. Coleoptiles of germinated seeds were placed in styrofoam and hydroponically cultured for 14 days. Then, 14-day old seedlings were transplanted into sterile and native soils, respectively. Native soils were the same as those used in the pot experiment. Sterilized soil was prepared by autoclaving. Plants were grown in an acclimatized room at 32/25°C day/night, 16 h light and 35% humidity. After 2 weeks, shoot lengths, total dry mass, leaf SPAD values and total nitrogen content for plants were measured.

### Metabolite fingerprinting analysis of root and rhizosphere soil

The roots selected for metabolite analyses were the same as root samples used for bacterial 16sRNA gene profiling. Freeze-dried roots were crushed for 1.5 min using a zirconia bead in a mixer mill (MM 400, Retsch). Then, 100 mg of the powder was weighted and extracted overnight at 4℃ using 0.6 ml of 70% aqueous methanol. After centrifugation at 10, 000 g for 10 min, extracts were passed through the SPE Cartridge (250 mg, 3 ml; CNW, ANPEL, Shanghai, China) and filtered via micro-pores (0.22 μm pore sizes; ANPEL, Shanghai, China) before UPLC-MS/MS analysis. Root metabolites were assayed by Ultra Performance Liquid Chromatography (UPLC, Shim-pack UFLC SHIMADZU CBM30A, Japan) coupled with Tandem mass spectrometry (Applied Biosystems 4500 QTRAP, USA). Root metabolites were determined as shown in Text S1. A widely targeted metabolomic method was used to profile root metabolites using the self-built MWDB database (Metware biotechnology Co., Ltd. Wuhan, China, http://www.metware.cn/). Secondary spectrum information was used to qualitatively analyze the metabolites. Further, triple quadruple-bar mass spectrometry was used for metabolite quantification.

In order to determine the metabolites released from roots, the metabolomes of rhizosphere soils from RC and WT were examined. The rhizosphere represents a key site for plant-microbe interactions within the soil. The composition of rhizosphere soil metabolites is intricate, encompassing root exudates, microbial metabolites, and the decomposition of plants, microbes, and organic matter within the soil. Compared to LC-MS, GC-MS offers distinct advantages in identifying complex metabolites. Therefore, GC-MS analysis was conducted for soil metabolic profiling of RC and WT rhizospheres. Rhizosphere soils were collected within 5 mm of root surface. Soil metabolite extraction was done as reported by Song et al. [43] (Text S2). Briefly, 2 g soil sample was placed in a 15 ml tube and supplemented with 3 ml of 75% methanol and 3 m of ethyl acetate. In this assay, 10 µL adonitol (10 mg/ml) was used as the internal standards. After homogenization with a 45-Hz ball mill for 1 min, samples were subjected to ultrasound for 10 min in ice water. Then, they were centrifuged for 10 min at 10,000 rpm and 4 °C. Their supernatants were transferred to 15 ml tubes and supplemented with a mixture of 3 ml 75% methanol and 3 ml ethyl acetate. The supernatants were subjected to ball milling and then filtered using 0.45 μm pore size (Millipore) and dried without heating in a vacuum concentrato. A solution of 100 L of methoxyamine (20 mg/ml in pyridine) hydrochloride was used to dissolve the samples under incubation at 37 °C for 2 h. Following this, 70 µL MSTFA was added and a 30-min trimethylsilylation reaction performed at 37 °C. The soil metabolome was analyzed by gas chromatography-mass spectrometry (GC-MS) using a Shimadzu GC-2010 plus equipped with a Shimadzu TQ8040 triple-quadrupole MS (Shimadzu, Kyoto, Japan). Soil metabolites were determined as shown in Text S2. Based on the GC-MS spectrum, soil metabolites were identified using the NIST MS search 2.0 from the National Institute of Standards and Technology (NIST). The concentrations of added internal standards were used to normalize the data matrix.

Orthogonal partial least squares discriminant analysis (OPLS-DA) was performed to visualize the high-dimensional data and determine the variation in root and soil metabolomics between RC and WT. OPLS-DA was applied after log transformation (log2) and mean centering of data. To avoid overfitting, a permutation test (200 permutations) was performed. Significantly regulated metabolites between groups were determined by VIP ≥ 1 and absolute Log2FC (fold change) ≥ 1 VIP values were obtained from OPLS-DA results, which also includes plots of score and permutation, and were generated using “MetaboAnalystR” in R. The identified metabolites were annotated using the KEGG compound database (http://www.kegg.jp/kegg/compound/). Then, annotated metabolites were mapped to the KEGG pathway database (http://www.kegg.jp/kegg/pathway.html). Pheatmap R package (version 1.0.12) was used to create heatmaps.

### Analysis of the effects of specific metabolites on rice plant and root-associated bacteria growth

Citric acid was used to study the effects of root metabolites on root-associated bacteria growth. Briefly, 250 g of soil for rice cultivation was filled in a plastic cup and mixed with 200 mL of 50 µmol/L citric acid solution. Then, abundances of the six root-associated bacterial strains were assayed by qPCR at 24 h, 48 h, 7 d and 14 d after citric acid treatment. Growth parameters of rice plants were analyzed on the 14th day after citric acid treatment. Six root-associated bacterial strains (those selected to design a SynCom) were used in this assay. The citric acid was filter sterilized and aseptically added to the 8-fold dilution of LB medium to final concentrations of 0 µmol/L, 1 µmol/L, 5 µmol/L, 20 µmol/L, 50 µmol/L and 100 µmol/L, respectively. Then, 10 µL activated bacterial liquid for each bacterial strains were added to each tube. The strains were cultured at 30° C for 10 h under 180 rpm in a constant-temperature oscillator. Then, 200 uL of the culture medium was added to a 96-well enzyme labeled plate and the absorbance determined at 600 nm. To study its effect on the growth of six root-associated bacteria strains in the natural soil environment, citric acid was also added to the soil that used to cultivate the rice.

## Results

### Overexpressed *OSCIPK2* in rice root promoted nitrogen uptake and plant growth under nitrogen-limiting conditions

To study differences in nitrogen uptake and utilization characteristics between RC and WT genotypes, four nitrogen fertilizer levels were set in field experiments. The yield of RC increased significantly by 11.3% and 5.75% under nitrogen inputs of 0 kg·ha^− 1^ and 75 kg·ha^− 1^, respectively, compared to WT, primarily due to an increase in effective panicles (Table S2). No significant difference was observed between RC and WT at nitrogen inputs of 150 kg·ha^− 1^ and 225 kg·ha^− 1^. The variation trend of nitrogen levels between RC and WT were consist with the change trend of yield. Compared with WT, the nitrogen levels in RC were significantly increased by 11.75% and 4.42% at nitrogen fertilizer level of 0 kg·ha^− 1^ and 75 kg·ha^− 1^, respectively (Table S3). A quadratic equation was employed to analyze the relationship between yield and nitrogen application rates. The correlation coefficient R^2^ of the quadratic equation surpassed 0.99, indicating a high degree of fit. Based on the quadratic equation, the theoretical yield of RC was higher than that of WT. Furthermore, a reduction of 10.45% in nitrogen supply did not result in a noticeable decrease in yield for RC when compared to WT (Table S4).

**Fig. 1:**
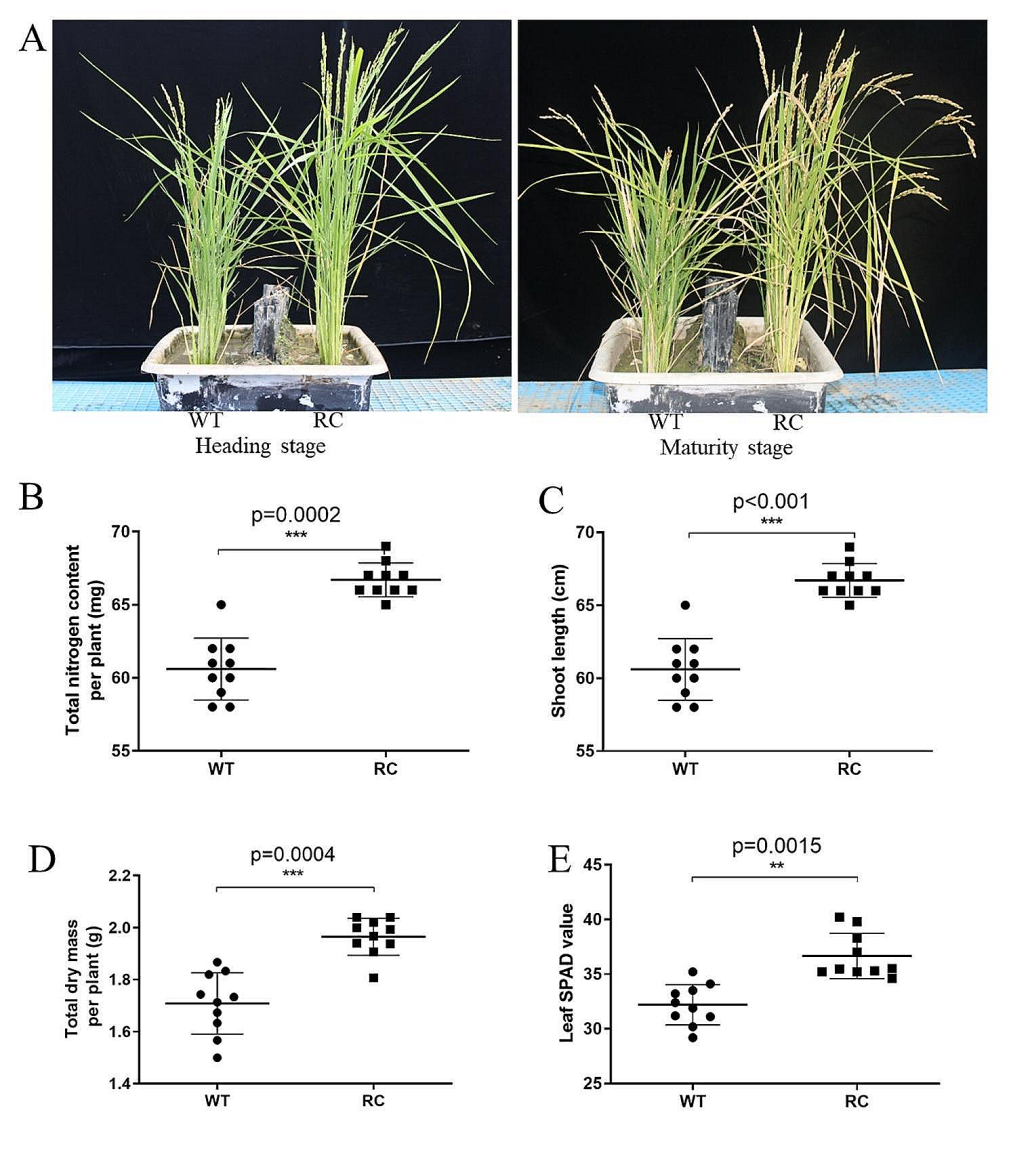
Comparisons between root-specific overexpressed *OSCIPK2* (RC) rice and wild type (WT) rice in low nitrogen soils. Fourteen days old rice seedlings of RC and WT were transplanted into the same plastic pot containing filed soils without nitrogen supplementation (low-nitrogen conditions). Plant growths were measured at heading stage and maturity stage. (**A**) Phenotypic comparisons of RC and WT at heading and maturity stages. (**B**) Total nitrogen levels of RC and WT at the heading stage, as measured by the Kjeldahl method. (**C**) Shoot lengths of RC and WT at heading stage. (**D**) Total dry mass per plant of RC and WT at heading stage. (**E**) Leaf SPAD value of RC and WT at heading stage, as measured by SPAD meter. *n* = 10, ***p < 0.001, **p-value 0.001 to 0.01

To mitigate the impact of field environment variables and facilitate sampling procedures, RC and WT were transplanted into plastic pots. The most significant disparity in yield between RC and WT was observed at the 0 kg·ha^− 1^ nitrogen level in the field experiment. Consequently, a nitrogen-free treatment (0 kg·ha^− 1^) was implemented in the pot experiment. RC exhibited superior performance compared to WT during both the heading and maturation stages (Fig. 1). Compared to WT, shoot length, total dry mass, leaf SPAD value and total nitrogen content of RC were increased by 10.06%, 15.68%,13.85% and 15.85% at the heading stage, respectively. At the maturation stage, yields were measured under low nitrogen conditions in pots, revealing that the yields of RC increased by 50.6% compared to WT (Table S5). This increase was significantly greater than the yield differences observed between RC and WT in the field experiment.

### RC and WT have distinct root microbiota in low nitrogen soils

To assess differences in root microbiota abundance between RC and WT in low nitrogen soils, bacterial community profile for root microbiota was created by amplification of the 16 S rRNA gene targeting the V5–V7 region using primers 799 F and 1193R, followed by Illumina sequencing. A total of 42,857 effective tags with bacterial species annotations were obtained from 6 root samples, with each providing an average of 7,142 effective tags. Rarefaction curves were created based on OTUs at 97% similarity (Fig. S1) and appeared to reach a plateau. At a 97% sequence similarity cut-off, 777 and 748 were obtained in RC and WT roots, respectively.

**Fig. 2:**
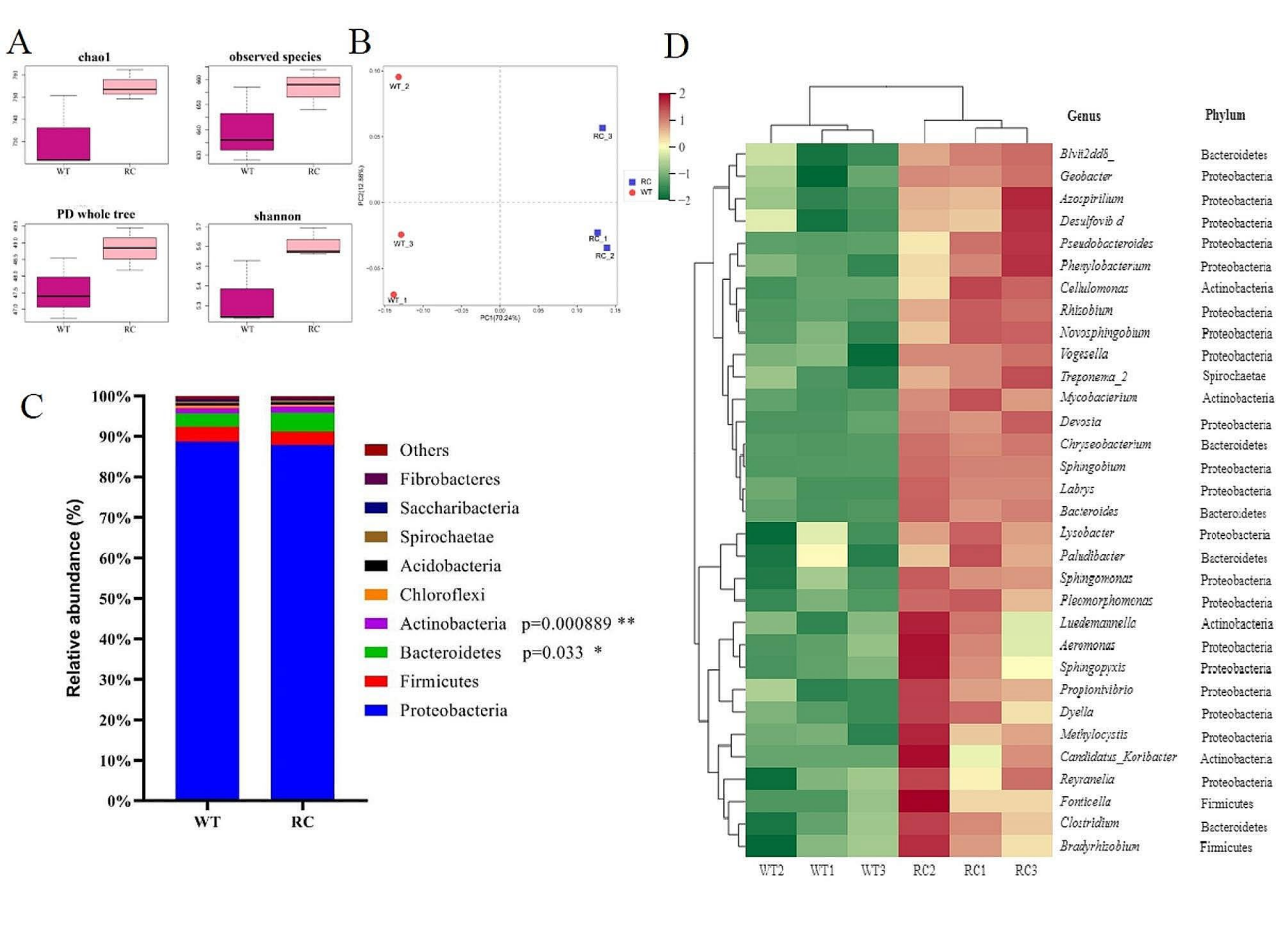
Root microbiome for root-specific overexpressed *OSCIPK2* (RC) rice and wild type (WT) rice. (**A**) Calculations of Shannon, Chao1, Observed_species and PD whole tree of root microbiome between RC and WT. (**B**) Principal coordinate analysis (PCoA) of bacterial communities based on weighted Unifrac algorithm for two different root samples. Centroids of Bray–Curtis dissimilarity scores for the composition of root sample compartments in the RC differed from WT. (**C**) Relative abundance, at phylum level, of the dominant bacteria in RC and WT rice root. (**D**) Heatmap depicting that relative abundances of OTUs were increased in RC, compared to WT. Heatmap was generated using Mothur (version 1.31.2) and was color-coded based on row z-scores

To elucidate on effects of plant genotypes on root microbial diversity, α- andβ- diversity analyses were performed. α-diversity was used to assess the complexity of species diversity for each sample. Compared to WT root, there were higher values of Shannon, Chao1, Observed species and PD whole tree in RC root (Fig. 2A) indicating that bacterial communities in RC root were significantly more diverse and complex than those of WT root. β- diversity were calculated to assess species complexity. In the principal coordinate analysis (PCoA) of Bray-Curtis similarities (β-diversity), the first two components (PC1 and PC2) of PCoA explained 70.24% and 12.86% of total bacterial community variations, respectively (Fig. 2B), implying clear differences in root bacteria composition between RC and WT.

**Fig. 3:**
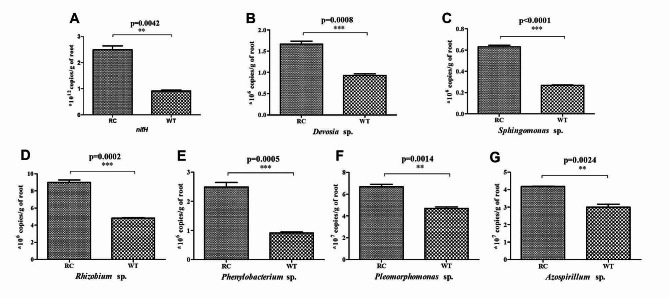
Quantification of the *nirH* gene and specific bacterial strains in the roots of root-specifically overexpressed *OSCIPK2* (RC) rice and wild type (WT) rice under the low nitrogen conditions. RNA was isolated from roots at the heading stage and used for qPCR. (**A**) Absolute abundance of *nirH* gene in the roots of RC and WT. (**B**-**G**) Absolute abundance of *Devosia* sp., *Sphingomonas* sp., *Rhizobium* sp., *Phenylobacterium* sp., *Pleomorphomonas* sp., and *Azspirillum* sp.in the roots of RC and WT. The absolute abundance of *nifH* and specifically bacterial strains in roots were quantified relative to a standard curve for plasmids containing the target gene or bacterial strains sequence inserts. Data are shown as mean **±** standard deviation (one-way analysis of variance, *n* = 3). ***p < 0.001, **p-value 0.001 to 0.01

To establish plant genotype-mediated differences in root bacterium structures, pairwise comparisons were performed using DEseq2. At the phylum level, OTUs in RC and WT roots were primarily assigned to the 10 bacterial phyla. The dominant phyla between RC and WT sample was Proteobacteria, accounting for 87.92% and 88.75% of bacterial sequences, respectively. In addition, Firmicutes, Bacteroidetes and Actinobacteria were also present in the two samples with relative abundances between 1% and 4%. The relative abundances of Bacteroidetes and Actinobacteria were found to be significantly higher in RC root compared to WT root in nitrogen-limiting soils (*p* < 0.05; Fig. 2C). A total of 59 genera with differing abundances between RC and WT roots were identified at the genus level (*p* < 0.05), primarily belonging to the proteobacteria phyla. Of these, 34 genera exhibited increased abundances in RC roots (Fig. 2D), while 25 genera showed decreased abundances (Fig. S2). Functional analysis indicated that 11 genera enriched in RC roots were associated with nitrogen-fixing capabilities. They included *Phenylobacterium* [44], *Rhizobium* [45], *Sphingomonas* [46], *Pleomorphomonas* [47], *Devosia* [48] and *Azospirillum* [49]. To estimate population sizes of nitrogen-fixing bacteria in RC roots, qPCR assays of root samples from RC and WT at the heading stage were performed. The results showed that the copy numbers (×10^12^/g of root) of *nifH* genes in RC roots were significantly greater than those in WT roots (Fig. 3A), suggesting that in nitrogen-deficient soil conditions, the nitrogen fixation capabilities of RC roots may be heightened compared to WT roots.

### Acquisition and quantification of specific bacterial strains in roots

In order to investigate the functions of specific root bacteria enriched in RC root, 16SrRNA sequences of 34 root bacteria with increased relative abundances in RC were identified by aligning them against sequences from the rice root bacterial culture collection established by Zhang et al. [33]. Twenty-two unique bacterial strains were successfully identified (sequence identity ≥ 97%), with 6 bacterial species demonstrating nitrogen fixation capabilities (species of the genera *Phenylobacterium*, *Rhizobium*, *Sphingomonas*, *Pleomorphomonas*, *Devosia* and *Azospirillum;* Table S6) selected for further analysis. Quantification of these root bacteria strains was performed by qPCR. First, the standard curve was established according to absolute copies and their Ct values, respectively. In Figure S3, a linear correlation with an R^2^ value exceeding 0.99 was observed when plotting the Ct values relative to absolute copies of six bacterial strains, indicating the suitability of the standard curve for absolute quantification. The levels of *Phenylobacterium sp*., *Rhizobium sp*., *Pleomorphomonas sp*., *Devosia sp*., *Sphingomonas sp.*, and *Azospirillum sp*. in RC roots showed enhancements of up to 2.48-, 1.85-, 1.42-, 1.79-, 1.39-, and 2.36-fold, respectively, compared to WT roots, consistent with results from deep pyrosequencing analysis (Fig. 3B-G).

### Effects of root-associated bacteria on rice growth

**Fig. 4:**
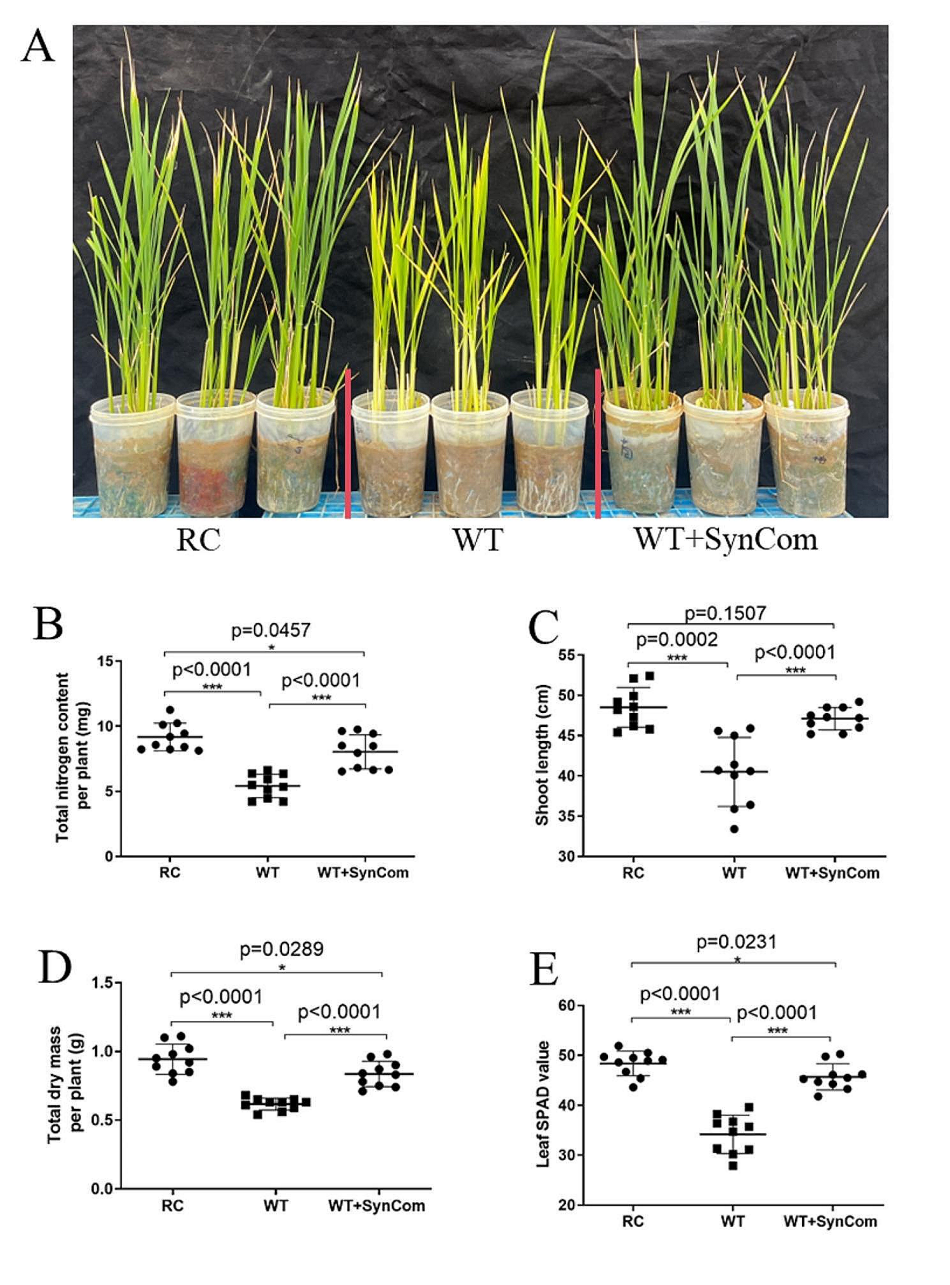
Synthetic microbial communities (SynCom) promoted rice growth in low-nitrogen soils. Fourteen days old rice seedlings were transplanted into plastic cups containing 250 g soil without nitrogen supplementation (low-nitrogen conditions). The soil was the same as in the pot experiment and mixed with SynCom, which was consisted of *Phenylobacterium* sp., *Rhizobium* sp., *Pleomorphomonas* sp., *Devosia* sp., *Sphingomonas* sp. and *Azspirillum* sp.,. After 14 days of SynCom treatment, the growth parameters were measured. (**A**) Rice growth after 14 days of SynCom treatment. (**B**) Total nitrogen content per plant, as measured by the Kjeldahl method. (**C**) Shoot length. (**D**) Total dry mass per plant. (**E**) Leaf SPAD value, as measured by SPAD meter. *n* = 10. ***p < 0.001, **p-value 0.001 to 0.01, *p-value 0.05 to 0.01

In the current study, the functions of root-associated bacteria were verified using the method of SynCom. A SynCom was designed using six bacterial strains (*Phenylobacterium* sp., *Rhizobium* sp., *Pleomorphomonas* sp., *Devosia* sp., *Sphingomonas* sp., and *Azospirillum* sp.) to assess their impact on rice growth in low nitrogen environments. After a 14-day co-cultivation period with SynCom in sterile soil, there were no apparent phenotypic differences observed between the SynCom treatment and control groups (Fig. S4). Furthermore, the native soil from the experimental field was specifically selected for the cultivation of rice plants. Following a 14-day co-cultivation period with SynCom in the native soil, significant improvements were noted in the total nitrogen levels, shoot length, total dry mass, and leaf SPAD values of the rice plants. These enhancements showed increases of 1.48- 1.08-, 1.16-, and 1.34-fold, respectively, compared to the control group. During a comparable growth period, RC transgenic rice demonstrated significant increases in total nitrogen levels, shoot length, total dry mass, and leaf SPAD values compared to the control group. Conversely, the SynCom group displayed notable decreases in total nitrogen levels, total dry mass, and leaf SPAD values when compared to RC, with no significant difference observed in shoot length (Fig. 4).

### RC and WT had distinct root metabolomes in low N soil

**Fig. 5:**
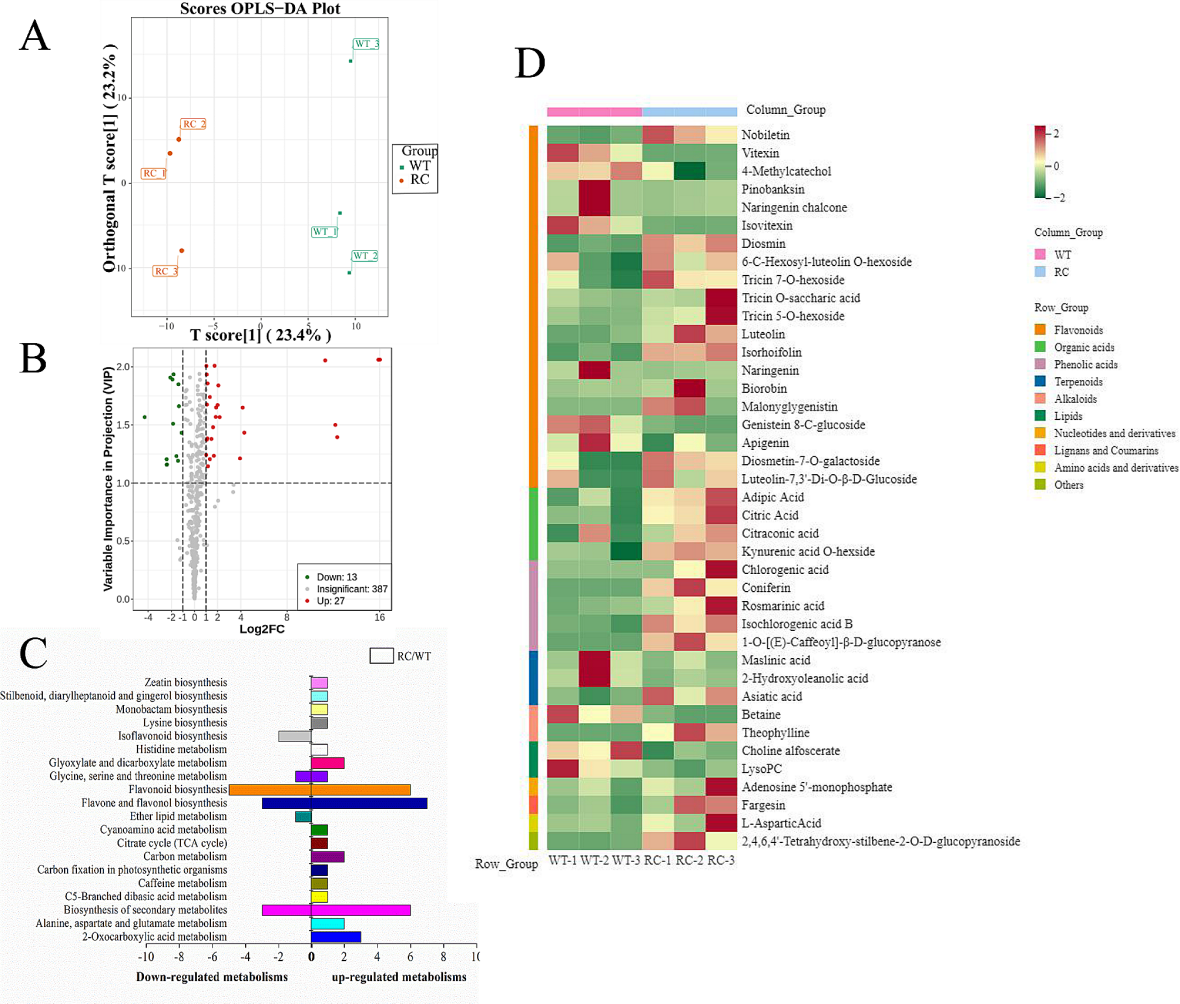
Metabolites fingerprinting of root metabolites of root-specific overexpressed *OSCIPK2* (RC) rice and wild type (WT) rice under nitrogen-limiting condition. (**A**) Orthogonal partial least squares-discriminant analysis (OPLS-DA) scores scatter plot for RC and WT based on root metabolic profiles. Data were obtained by metabolite fingerprinting (UPLC-MS/MS analysis in positive and negative ion mode and controlled by Analyst 1.6.3 software). (**B**) VIP (Variable importance in the project)-plot of the OPLS-DA model. Root metabolites with VIP scores ≥ 1 were considered to be significantly differentially expressed between RC and WT. (**C**) Metabolic pathway analysis of the identified differentially expressed metabolites. Root metabolites were identified by searching by the self-built database, MWDB (Metware biotechnology Co., Ltd. Wuhan, China) (http://www.metware.cn/). Identified metabolites were annotated and mapped to KEGG database (http://www.kegg.jp/kegg). UP-and Down-regulated metabolites indicated increased or decreased metabolite levels in RC relative to WT. (**D**) Heatmap analysis of differentially expressed root metabolites between RC and WT. High and low metabolite levels are represented as reddish and greenish scales, respectively. Heatmaps were created using the pheatmap R package (version 1.0.12)

Metabolomic profiling via LC-MS analysis identified 427 root metabolites in RC and WT plants grown in low nitrogen soils. OPLS-DA analysis revealed significant differences in root metabolomes between the two (Fig. 5A), suggesting distinct metabolic strategies for nitrogen adaptation. Metabolites with VIP values ≥ 1.0 and *p* < 0.05 were considered significantly change. The OPLS-DA model revealed 40 significantly differentially expressed metabolites (Fig. 5B, Table S7), with 27 up-regulated and 13 down-regulated in RC roots (Fig. 5C). The enrichment analysis showed that differential metabolites mainly involve in the biosynthesis of flavonoids and organic acids (Fig. 5D). The findings revealed that roots of the RC genotype exhibited elevated concentrations of organic acids, such as adipic acid, citric acid, and citraconic acid, in comparison to roots of the WT genotype. This suggests the significant contribution of organic acids in facilitating the response of rice roots to low nitrogen conditions.

### RC and WT had distinct rhizophere soil metabolomes in low N soil

**Fig. 6:**
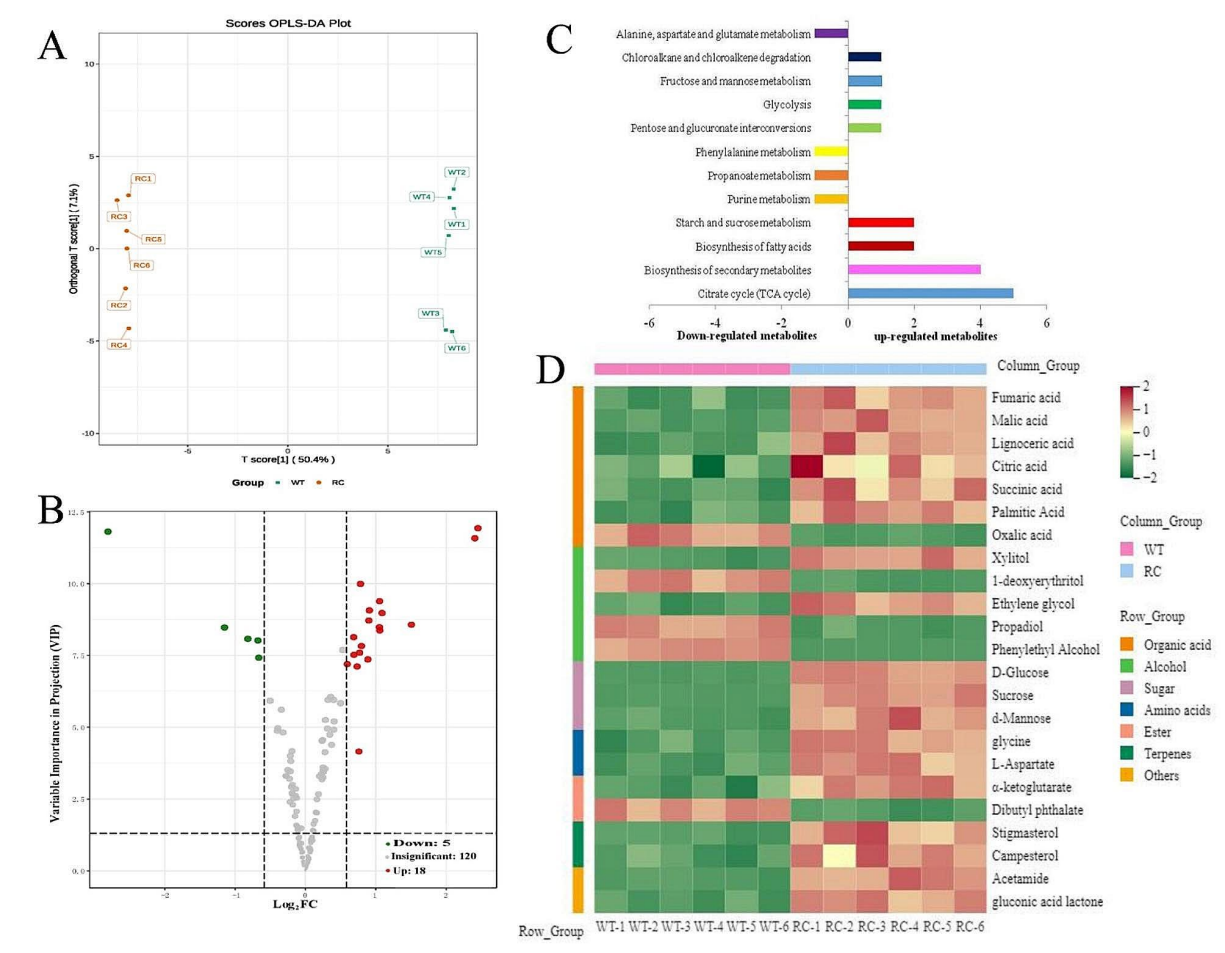
Metabolites fingerprinting of rhizosphere soil metabolites of root-specific overexpressed *OSCIPK2* (RC) rice and wild type (WT) rice under nitrogen-limiting conditions. (**A**) Orthogonal partial least squares-discriminant analysis (OPLS-DA) scores scatter plot for RC and WT based on soil metabolic profiles. Data were obtained by metabolite fingerprinting of GC-MS. (**B**) VIP (Variable importance in the project)-plot of the OPLS-DA model. Soil metabolites with VIP scores ≥ 1 were considered to be significantly differentially expressed between RC and WT. (**C**) Metabolic pathway analysis of the identified differentially expressed metabolites. Soil metabolites were identified by searching in National Institute of Standards and Technology (NIST) library (NIST Version 14.0). the identified metabolites were annotated and mapped to KEGG database (http://www.kegg.jp/kegg). UP-and Down-regulated metabolites indicated increased or decreased metabolite levels in RC, relative to WT. (**D**) Heatmap analysis of differentially expressed soil metabolites between RC and WT. High and low metabolite levels are presented as reddish and greenish scales, respectively. Heatmaps were created using the pheatmap R package (version 1.0.12)

Metabolomic profiling using GC-MS analysis revealed the presence of 143 soil metabolites in the rhizospheres of RC and WT plants grown in a nitrogen-deficient environment (Fig. 6A). Of these metabolites, 23 exhibited significant differences, with 18 showing up-regulation and 5 down-regulation in RC compared to WT. Specifically, organic acids such as fumaric acid, malic acid, and citric acid were found to be more prevalent in the rhizospheres of RC plants. Additionally, alcohols emerged as the second group of metabolites displaying differential expression between the rhizosphere soils of RC and WT plants. Three sugars (D-glucose, sucrose, D-mannose) were significantly higher in RC rhizosphere compared to WT (Fig. 6D). KEGG analysis revealed enrichment in citrate cycle, secondary metabolites biosynthesis, fatty acid biosynthesis, and starch/sucrose metabolism in differentially expressed metabolites in rhizosphere soils. Of particular significance, the critical intermediate citric acid in the citrate cycle exhibits an increasing trend in both the rhizosphere soil and root system of RC. This suggests the citric acid may play an important role in assembly of root microbiome under low nitrogen environments.

### Effects of citric acid on growth of rice plant and root-associated bacteria

**Fig. 7:**
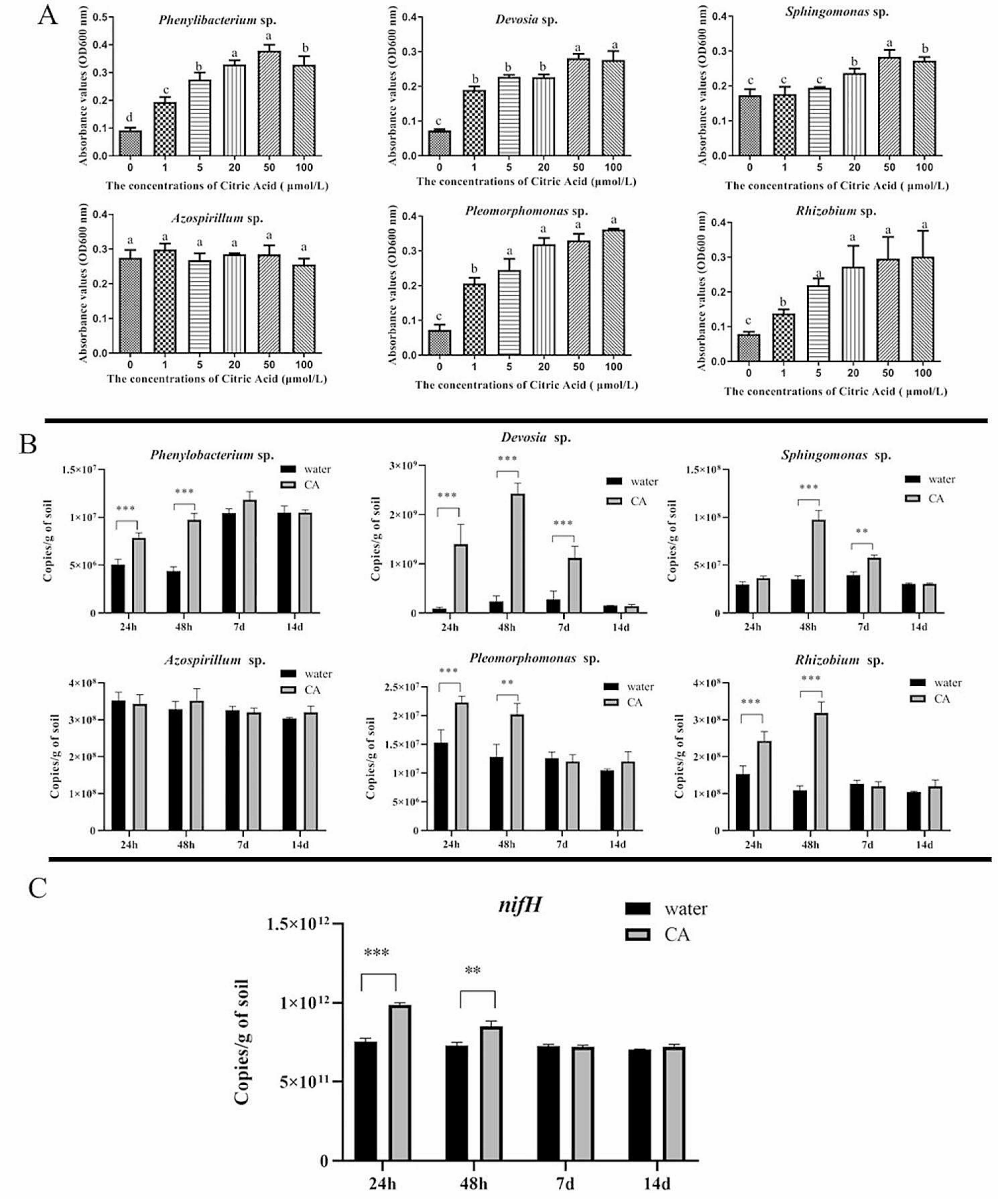
Effects of citric acid on growth of root-associated bacteria. (**A**) Effects of increasing citric acid concentrations (0,1, 5, 20, 50 and 100 µmol/L) on the growth of root-associated bacteria in LB medium. Bacterial growth is presented as absorbance value at OD 600 nm. (**B**) Effects of 50 µmol/L citric acid on root-associated bacteria abundance in the low nitrogen soils. (**C**) Effects of 50 µmol/L citric acid on absolute abundance of *nifH* gene in the low nitrogen soils. Citric acid was directly applied to the soil cultivated with 14 days old rice seedlings. Soil samples were collected at 24 h, 48 h,7 and 14 days after citric acid treatment, and used for the DNA extraction and qPCR. The absolute abundances of *nifH* and root-associated bacterial in soil were quantified relative to a standard curve for plasmids containing the target gene or bacterial strains sequence inserts. Columns with different letters are statistically different (LSD test, *p* < 0.05). ***p < 0.001, **p-value 0.001 to 0.01

The present study examines the effects of citric acid on the growth of root microbiota in LB medium. Six root-associated bacteria strains, which were selected to design a SynCom, were used in this experiments. The findings indicate that the biomasses of *Phenylobacterium* SP., *Rhizobium* SP., *Pleomorphomonas* SP., *Devosia* SP. and *Sphingomonas* SP. gradually increased citric acid levels from 0 to 50 µmol/L. At 100 µmol/L, no additional growth in biomass was observed. (Fig. 7A). Furthermore, citric acid (50 µmol/L) was directly added into the low nitrogen soil cultivated with WT rice. However, there were no obvious differences in growth of rice plant in low nitrogen soils on the 14th day after citric acid treatment (Fig. S5). Quantitative PCR analysis demonstrated a significant increase in the abundance of five bacterial strains in soil, namely *Phenylobacterium* SP., *Rhizobium* SP., *Pleomorphomonas* SP., *Devosia* SP., and *Sphingomonas* SP., within 48 h following citric acid treatment (Fig. 7B). However, by the 14th day, the differences in abundance of all selected bacterial strains between the citric acid and water treatment groups were found to be statistically insignificant. Furthermore, analyses of variations in *nifH* abundance in response to citric acid treatment were conducted, revealing significant differences in gene copy numbers within 48 h, while differences in gene copy numbers at days 7 and 14 were deemed insignificant (Fig. 7C).

## Discussion

The advancement of cultivars with high nitrogen use efficiency (NUE) or low nitrogen tolerance is essential for the progression of sustainable agriculture. Nitrogen uptake and transport efficiency are influenced by the plant species and genotypes. A prior investigation demonstrated that the overexpression of *OSCIPK2* in rice roots (RC) notably enhanced nitrogen uptake in low-nitrogen soils [32]. The findings of this study indicate that a reduction of 10.45% in nitrogen supply did not lead to a significant decrease in yield for RC compared to WT in an open-field system. Furthermore, the study observed higher yields for RC in the pot system compared to the field system, potentially due to lower total soil nitrogen levels in the pot system relative to the field system. These findings suggest that *OSCIPK2* is a gene associated with low nitrogen tolerance in rice plants, facilitating increased nitrogen absorption from soils under conditions of nitrogen deficiency. Recent research has demonstrated a strong correlation between nitrogen uptake in crops and the composition of the root microbiome [33, 50]. Plants attract root microbiota from the surrounding soil through the release of root exudates. The presence of root microbiota expands the ecological niche and metabolic capabilities of plants, enhancing nutrient uptake, promoting plant growth, and improving responses to environmental stressors [51, 52]. The current investigation utilized a multi-omics approach, integrating microbiome sequencing with metabolomics, to demonstrate that *OSCIPK2* induces substantial changes in the composition of root-associated bacterial communities and metabolite profiles in root and rhizosphere soils under conditions of nitrogen limitation. This study provides evidence supporting the notion that interactions between genotype and microbiome influence the phenotype of plants [53].

High-throughput sequencing analysis demonstrated a clear distinction in root bacterial composition between the RC and WT plants, suggesting a significant impact of *OSCIPK2* on root bacteria establishment in nitrogen-limited environments. Previous studies have shown that plant genotypes significantly influence the composition of root-microbiome interactions. For instance, the root-specific transcription factor MYB72 plays a crucial role in regulating the excretion of coumarin scopoletin, a phenolic compound that aids in iron mobilization and exhibits antibacterial properties against certain beneficial microbial communities associated with roots [54]. Additionally, another study has shown that genes responsible for phosphate starvation responses facilitate the recruitment of root microbiomes necessary for efficient phosphate absorption by plant roots [55]. Subsequent analysis demonstrated greater diversities in root microbiota associated with RC compared to WT cultivars, suggesting that RC roots harbored a larger number of bacterial species. Recent studies on rice have also shown that the increased microbial diversities in *indica* cultivars compared to *japonica* cultivars contribute to the high nitrogen use efficiency (NUE) in *indica* [33]. Thus, a high microbial diversity in rice roots may play a significant role in nitrogen uptake and utilization efficiency.

Genus-level analysis revealed that *Phenylobacterium, Rhizobium, Sphingomonas, Pleomorphomonas, Devosia* and *Azospirillum* were the predominant microbiota present in RC root. Notably, these genera have been previously documented to possess nitrogen-fixing capabilities. *Rhizobium* is a well-known nitrogen-fixing bacterium in legumes and rice roots [56], while other bacteria like *Azospirillum* [57], *Pleomorphomonas* [58], and *Sphingomonas* [46] are also associated with nitrogen fixation in rice roots. *Phenylobacterium* [44] and *Devosia* [48] genera have been reported to have nitrogen-fixing capacities in other crops. These results indicated that the capabilities of nitrogen fixation in RC root enhanced in the low nitrogen soil, which was also confirmed by increased copies of the *nifH* gene. In the context of rice cultivation, nitrogen-fixing bacteria have been found to contribute between 36 and 42% of the plant’s nitrogen requirements [59, 60]. This study examined the abundance of six bacterial strains belonging to nitrogen-fixing genera in rice roots, revealing a significantly higher presence of these strains in RC roots compared to WT. The findings suggest that the overexpression of *OSCIPK2* in rice roots promotes the colonization of nitrogen-fixing bacteria, thereby enhancing their nitrogen-fixing capacity and enabling RC plants to better assimilate nitrogen in nitrogen-deficient environments compared to WT plants. Notably, in the field experiment, it was observed that total nitrogen levels in RC plants exhibited a significant increase only in low nitrogen conditions, suggesting that high nitrogen may inhibit their nitrogen-fixing abilities. This is similar to the “nitrogen repression” effect seen in legumes.

To determine the roles of bacteria present in RC roots, the SynCom experiment was conducted. SynCom serves as a valuable tool for studying the factors that facilitate the formation of microbial communities, their regulatory functions, and the molecular mechanisms linked to plant growth and development [61]. Our findings revealed the presence of six nitrogen-fixing bacterial strains within the SynCom, which notably enhanced the growth of rice in nitrogen-deficient soils, underscoring the significance of bacteria inhabiting RC roots in facilitating plant nitrogen absorption and growth. Significantly, when SynCom was utilized in sterile soils, no discernible phenotypic distinctions were observed between the SynCom treatment and control groups. Previous research often focused on plant varieties cultivated under largely sterile conditions, yet the presence of a native microbiome in field settings suggests a non-sterile environment. Inoculating rhizosphere soil with external strains has been shown to enhance plant growth by activating local bacterial species with known plant growth-promoting properties [62–64]. Our research also revealed that the six bacterial strains comprising SynCom have the potential to act as “hub microorganisms” in facilitating the assembly of the RC root microbiome. These key species can significantly impact microbial community structures through robust biotic interactions with the host plant or other microorganisms, rather than solely due to their own numerical dominance [65, 66]. The presence of hub microorganisms can exert direct and indirect influences on microbiome assembly and facilitate interactions between the plant and its associated microbial community [67]. Future studies should explore the effects of these hub microorganisms on the assembly of the RC root microbiome under nitrogen-limiting conditions. Furthermore, in this research, the six root-associated bacterial strains were combined into a SynCom in equal quantities. However, for subsequent investigations, it would be more ecologically valid to construct the SynCom based on the relative abundances of these six bacterial strains as identified in the soil or root sample through qPCR, thereby providing a more accurate representation of the bacterial community composition. In addition, it is noteworthy that while SynCom treatment can indeed enhance rice growth to some extent in low-nitrogen soils, there remains a disparity compared to RC transgenic plant. Perhaps this discrepancy arises from the fact that certain beneficial bacteria aiding nitrogen absorption in the root system of RC have yet to be isolated and identified in the present study.

Plants use ∼ 20% of their photosynthesized carbon to make root-derived organic molecules, which stimulate the formation of distinct root microbiota from the surrounding soils [54, 68, 69]. A study in *Arabidopsis* showed that triterpenes produced by roots can shape the root-associated microbiome [70]. A total of 40 and 23 differentially expressed metabolites were identified in root and rhizosphere soils between RC and WT, indicating that *OSCIPK2* regulates the synthesis and secretion of root metabolites in response to low nitrogen stress. Under a low nitrogen environment, organic acids were the main differential metabolic categories both in the rhizosphere soil and root between RC and WT. The organic acids secreted by plant roots can serve as carbon and nutrient sources for the growth of soil microorganisms and play regulatory roles in plant–soil–microbe interactions [71, 72]. Previous studies involving Tartary buckwheat [73], maize [74] and rice [75] showed that organic acids exuded by roots are beneficial to plants under low nitrogen stress by regulating soil nutrient availability. Plants secrete organic acids to recruit beneficial microbes in the root zone for coping with changes in inorganic nitrogen levels in rhizosphere soils [76]. Importantly, we found that under a low nitrogen environment, citric acid levels were significantly increased in both root and rhizosphere soils of RC in comparison with WT. Citric acid, a key component in the citrate cycle, is synthesized through the enzymatic action of citrate synthase, which combines acetyl-CoA and oxaloacetate. CIPKs, a novel class of plant Ca^2+^ sensors, have been identified as playing a significant role in response to various environmental stresses [77]. It is understood that the activity of CIPKs can be regulated by Ca^2+^ interacting with citrate synthase [78, 79]. Consequently, it is hypothesized that CIPKs may influence the production of citric acid by modulating intracellular Ca^2+^ levels in plants. The *OsCIPK17* gene in rice has been demonstrated to enhance rice drought resistance and play a role in the citric acid accumulation within the citrate cycle [80]. Ca^2+^ have the potential to enhance soybean tolerance to aluminum ions (Al^3+^) by stimulating citric acid secretion and mitigating oxidative stress damage [81]. Nevertheless, further investigation is required to elucidate the precise mechanisms by which *OsCIPK2* modulates citric acid biosynthesis in response to low nitrogen conditions.

In a previous study, some specific beneficial bacterial species, such as those known for promoting crop growth, were observed to be negatively impacted by citric acid [69]. For example, citric acid was identified as a chemotactic attractant for Pseudomonas fluorescens WCS365, a strain capable of establishing itself on tomato roots [82]. Likewise, watermelon roots release citric acid, which facilitates the colonization of roots by the growth-promoting rhizobacteria, *Paenibacillus polymyxa SQR-21* [83]. In the present study, the application of a concentration of citric acid (50 µmol/L) was found to enhance the growth of *Phenylobacterium* SP., *Rhizobium* SP., *Pleomorphomonas* SP., *Devosia* SP., and *Sphingomonas* SP., known for their nitrogen-fixing capabilities. Furthermore, the abundances of these bacterial strains in soil were significantly elevated at 24 h and 48 h post citric acid treatment. Gene copy numbers of *nifH* in soil revealed the same trend as the abundance change of the above 5 bacterial strains. These findings suggest that optimal levels of citric acid may play a crucial role in enhancing the nitrogen-fixing abilities of RC rice in low nitrogen soils. A study on the intercropping of maize and alfalfa demonstrated a positive correlation between atmospheric nitrogen levels and citric acid concentrations in the rhizosphere of alfalfa [84]. This relationship may facilitate the mobilization of soluble resources for nitrogen fixation in the absence of additional nitrogen inputs. There were no changes in the abundance of above 5 nitrogen-fixing bacterial strains in the soil on days 7 and 14 after citric acid treatment. This resulted in no significant differences in plant growth parameters, including total nitrogen levels at 14 days in low nitrogen soils. The complexity of soil microorganisms in the rhizosphere and rapid utilization or absorption of citric acid by other microbial communities may have contributed to this outcome. These findings suggest that sustained-release technologies should be taken into account when applying essential metabolites to soil in order to facilitate the development and proliferation of nutrient-related microorganisms.

## Conclusion

Enhanced *OSCIPK2* expressions in roots could promote citric acid production by rice to recruit nitrogen-fixing bacteria in its root from bulk soil in response to low-nitrogen stress. The use of SynCom made from RC root-enriched bacteria could significantly improve plant nitrogen acquisition in low-nitrogen soil. These results demonstrate that plant genotype has a strong effect on root microbiome diversity and abundance and influences plant nitrogen acquisition. However, our study focused on a specific set of root-associated bacterial strains and a particular rice variety under nitrogen-limitation conditions. Future studies could explore the interactions between different rice varieties and their associated root microbiomes. Such research would provide a more comprehensive understanding of the intricate relationships between plants and their associated microbial communities, paving the way for the development of more effective and sustainable agricultural practices.

### Electronic supplementary material

Below is the link to the electronic supplementary material.

Supplementary Material 1

## Data Availability

Data is provided within the manuscript or supplementary information files.

## Abbreviations

RC: Root-specific overexpressed *OSCIPK2*
SynCom: Synthetic microbial communities
NUE: Nitrogen use efficiency
CIPK: CBL-interacting protein kinases
OTUs: Operational taxonomic units
qPCR: Quantitative real-time PCR
PCA: Principal component analysis
VIP: Variable importance in the project
