# Supplementary material for: OsCIPK2 mediated rice root microorganisms and metabolites to improve plant nitrogen uptake
Source: BMC Plant Biol. 2024 Apr 16;24:285. doi: 10.1186/s12870-024-04982-0 (PMC11020999; doi:10.1186/s12870-024-04982-0)
Supplement: Supplementary file 1 — Supplementary Material 1 [file 12870_2024_4982_MOESM1_ESM.doc]

**Supplementary Material**

Figure S1: Rarefaction curves of bacterial communities based on observed OTUs at 97% sequence similarity for individual samples. RC and WT represent root-specific overexpressed *OSCIPK2* rice and wild-type rice. The number followed by the genotype represents the three replicates.

Figure S2: Heatmap showing that the relative abundances of OTUs were decreased in RC, compared to WT. Heat map was color-coded based on row z-scores. RC and WT represent root-specific overexpressed *OSCIPK2* rice and wild-type rice.

Figure S3: Standard curves for *nirH* gene and six root-associated bacteria.

Figure S4: The effects of synthetic microbial communities (SynCom) on the rice growth in the sterile soil with low nitrogen. Sterilized soil was prepared by autoclaving. The soil was mixed with SynCom, which consisted of *Phenylobacterium* sp.*, Rhizobium* sp.*, Pleomorphomonas* sp.*, Devosia* sp.*, Sphingomonas* sp. and *Azspirillum* sp.

Figure S5: The effects of the citric acid (50 μmol/L) on the rice growth in the low nitrogen soils. (A) Rice growth after 14 days of citric acid treatment. (B) Total nitrogen levels per plant, as measured by the Kjeldahl method (n=10). (C) Shoot length (n=10). (D) Total dry mass (n=10). (E) Leaf SPAD value, as measured by SPAD meter. (n=10).

Table S1: q-PCR primers for specific root-associated bacterial strains.

Table S2: Grain yields and components of RC and WT in field experiment under the low nitrogen conditions.

Table S3: Total nitrogen levels of root-specific overexpressed *OSCIPK2* (RC) rice and wild type (WT) rice under different nitrogen treatments.

Table S4: Nitrogen efficiency equation and nitrogen applications of the highest yield.

Table S5: Grain yields and its components from RC and WT in pot experiments under the low nitrogen conditions.

Table S6: The ID of nitrogen-fixing bacteria in rice roots. (A) Sequence identity coverage; (B) The ID of ACCC (Agricultural Culture Collection of China, https://www.iarrp.cn/en/aboutus/structure/scientific/287900.htm); (C) The ID of rice root bacterial culture collection.

Table S7: Screening results of differentially expressed root metabolites in RC, compared to WT. RC and WT represent root-specific overexpressed *OSCIPK2* rice and wild-type rice.

Table S8: Screening results of differentially expressed soil metabolites in RC rhizospheres, compared to WT rhizospheres. RC and WT represent root-specific overexpressed *OSCIPK2* rice and wild-type rice.

Text S1: Mass spectrometer parameters of LC-MS.

Text S2: Mass spectrometer parameters of GC-MS.

**Supplementary Figure:**

Figure S1


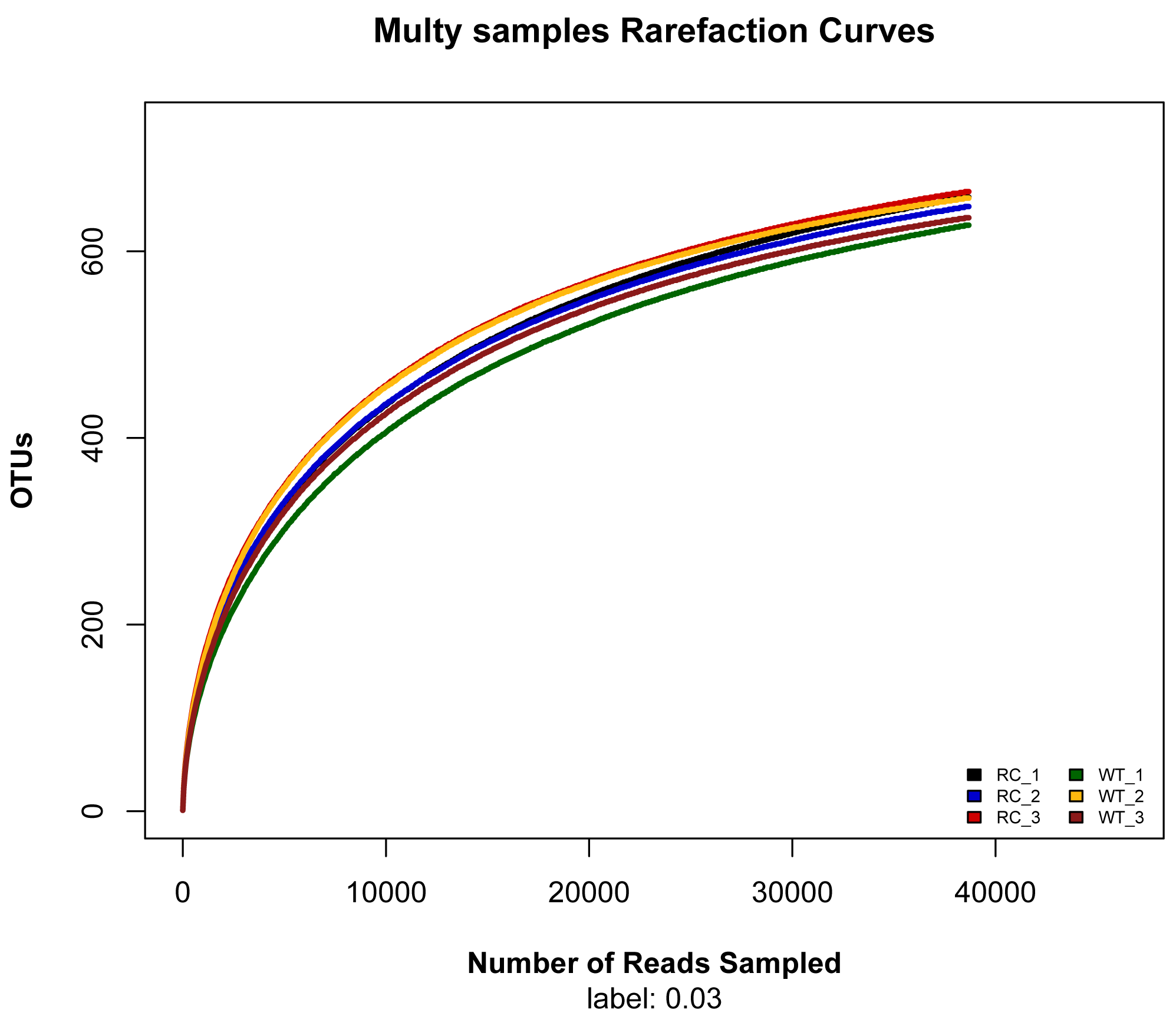


Figure S1: Rarefaction curves of bacterial communities based on observed OTUs at 97% sequence similarity for individual samples. RC and WT represent root-specific overexpressed *OSCIPK2* rice and wild type rice. The number followed by the genotype represents the three replicates.

Figure S2


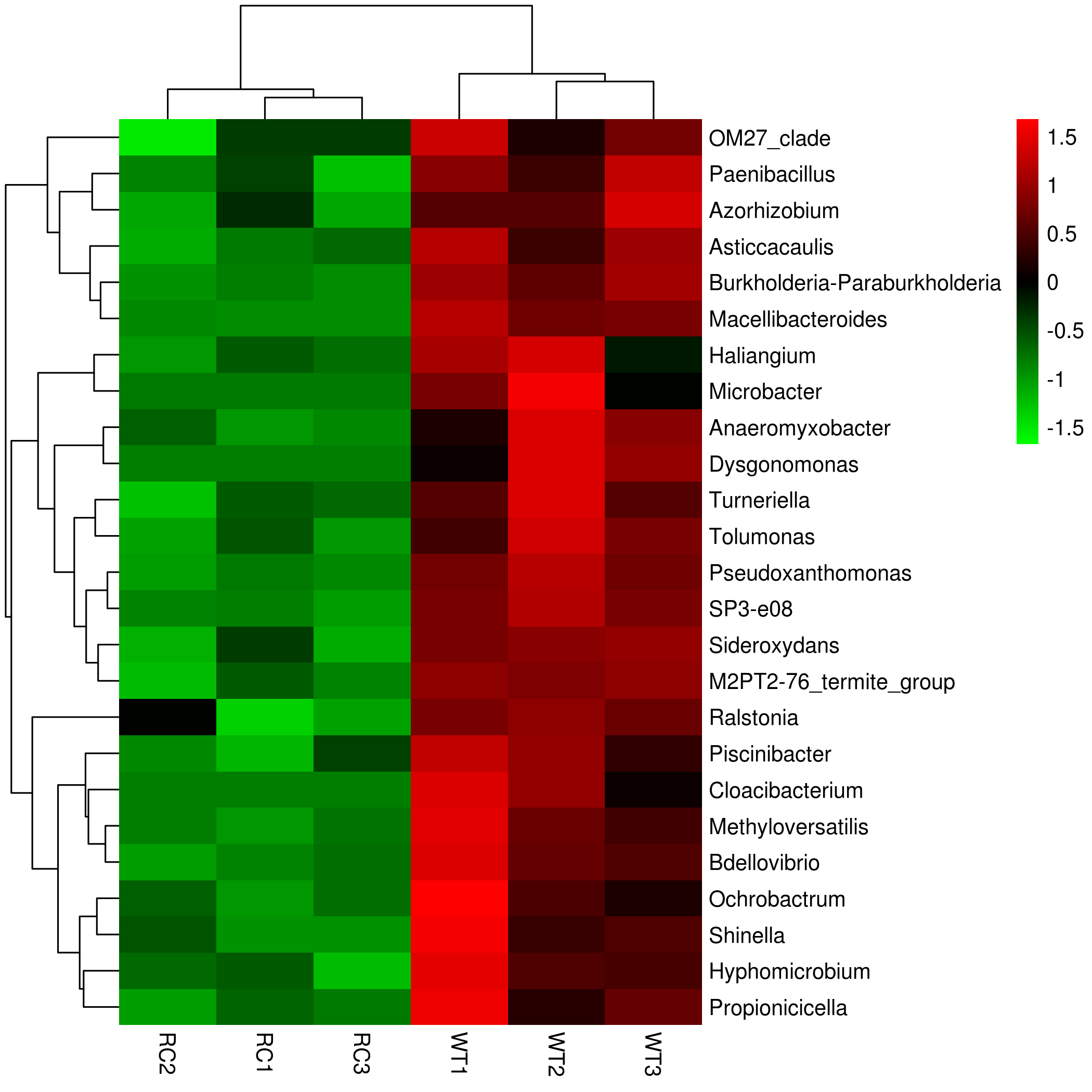


Figure S2: Heatmap showing that the relative abundances of OTUs were decreased in RC, compared to WT. Heat map was color-coded based on row z-scores. RC and WT represent root-specific overexpressed *OSCIPK2* rice and wild type rice.


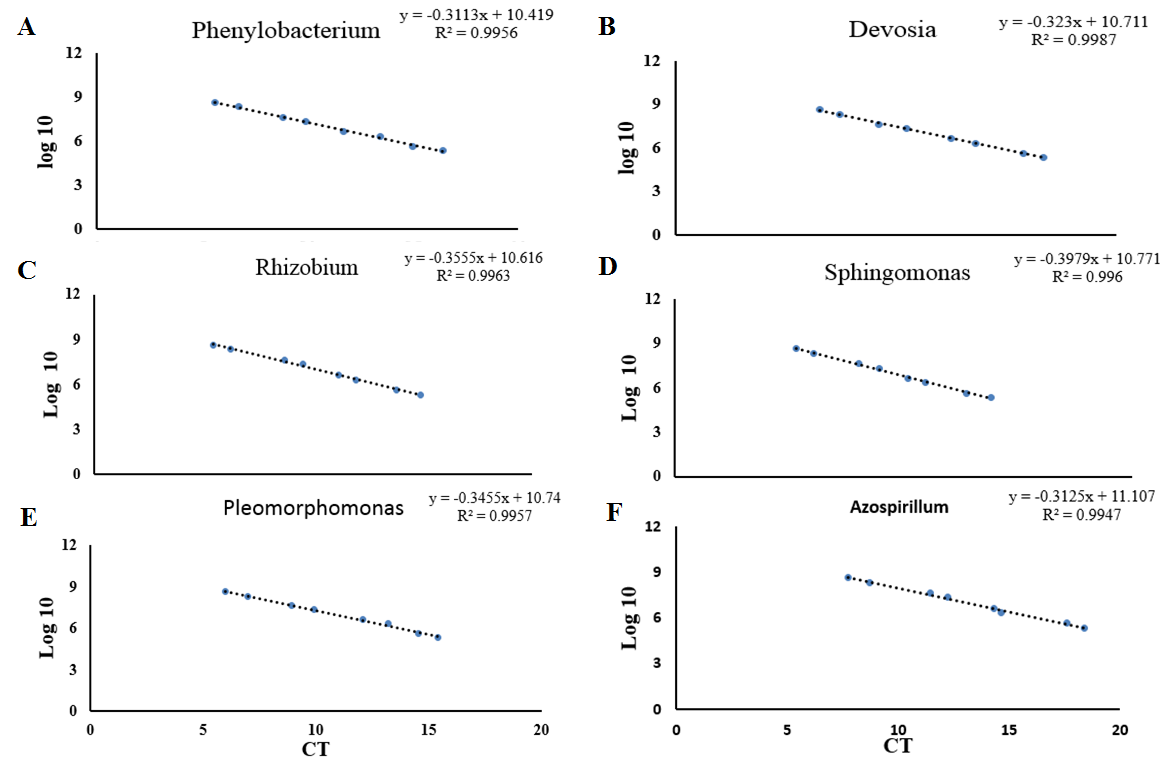
Figure S3

Figure S3: Standard curves for *nirH* gene and six root-associated bacteria.

Figure S4


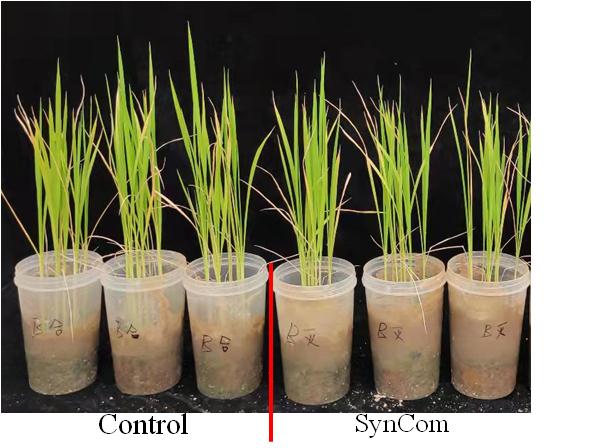


Figure S4: The effects of synthetic microbial communities (SynCom) on the rice growth in the sterile soil with low nitrogen. Sterilized soil was prepared by autoclaving. The soil was mixed with SynCom, which consisted of *Phenylobacterium* sp.*, Rhizobium* sp.*, Pleomorphomonas* sp.*, Devosia* sp.*, Sphingomonas* sp. and *Azspirillum* sp.

Figure S5


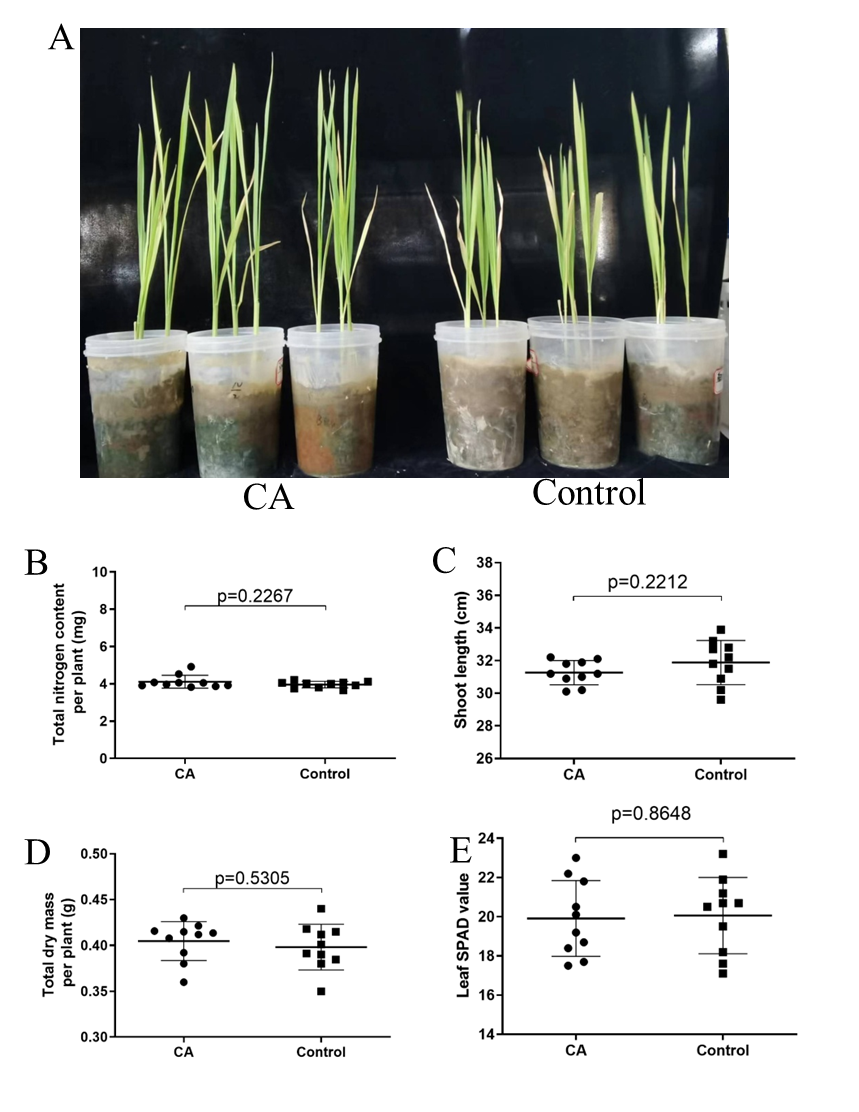


Figure S5: The effects of the citric acid (50 μmol/L) on the rice growth in the low nitrogen soils. (A) Rice growth after citric acid treatment. (B) Total nitrogen levels per plant, as measured by the Kjeldahl method (n=10). (C) Shoot length (n=10). (D) Total dry mass (n=10). (E) Leaf SPAD value, as measured by SPAD meter. (n=10).

Table S1 The qPCR primer for the specific root-associated bacteria strains

| Bacteria strain | Up | Down |
| --- | --- | --- |
| *Phenylobacterium* SP. | AGCACTCATCGTTTACAGCG | TATTCGGCGGAACACCAG |
| *Rhizobium* SP. | ACTGTCACCACCATTGTAGCAC | AGAACCTTACCAGCCCTTGA |
| *Sphingomonas* SP. | AACGCTGGTAAGGTTCTGC | GGACTGGTATTGACGCTGAG |
| *Pleomorphomonas* SP. | TCACCGCCATTGTAGCAC | CAGAACCTTACCAGCCCTTG |
| *Devosia* SP. | TCACCGCCATTGTAGCAC | GGAGACGGATTCCTTCACTT |
| *Azospirillum* SP. | TCAGGTAAAGCCAACTCCCA | GCGAATCTCCAAAAGCCATC |

Table S2 Grain yields and components of root-specific overexpressed *OSCIPK2* (RC) rice and wild type (WT) rice in field experiment under the low nitrogen conditions.

| N application rate (kg·Ha^-1^) | Genotype | Valid panicles (m^-2^) | Grains per panicle | Seed setting rate (%) | 1000-grain weight (g) | Yield (kg·Ha^-1^) |
| --- | --- | --- | --- | --- | --- | --- |
| 0 | WT | 424.27e | 35.53b | 93.16a | 25.33a | 3560.4f |
|  | RC | 457.33d | 36.73b | 93.53a | 25.22a | 3963e |
| 75 | WT | 474.73c | 40.27a | 94.16a | 25.34a | 4563.45d |
|  | RC | 493.00b | 41.00a | 93.79a | 25.44a | 4825.65c |
| 150 | WT | 512.43a | 41.67a | 94.58a | 25.66a | 5183.25a |
|  | RC | 522.00a | 41.80a | 94.37a | 25.55a | 5262.9a |
| 225 | WT | 506.05a | 41.00a | 95.35a | 25.43a | 5033.4b |
|  | RC | 508.37a | 40.80a | 94.88a | 25.37a | 4995.45b |

Different small letters in the same column mean significant difference at the 5% level.

Table S3 Total nitrogen levels of root-specific overexpressed *OSCIPK2* (RC) rice and wild type (WT) rice under different nitrogen treatments.

Different small letters in the same column mean significant difference at the 5% level.

| N application rate (kg·Ha^-1^) | Genotype | Total nitrogen content  per plant(mg) |
| --- | --- | --- |
|  |  |  |
| 0 | WT | 294.58f |
|  | RC | 329.57e |
| 75 | WT | 363.09d |
|  | RC | 379.02c |
| 150 | WT | 431.61b |
|  | RC | 437.99ab |
| 225 | WT | 441.57ab |
|  | RC | 446.98a |

Table S4 Nitrogen efficiency equation and nitrogen application of the highest yield

| Genotype | Nitrogen efficiency equation | highest yield (kg·ha^-1^) | | | N application rate of highest yield (kg·ha^-1^) |
| --- | --- | --- | --- | --- | --- |
| WT | y= -0.7686x^2^+18.247x+236.07 , r^2^=0.9954 | | 5165.55 | 178.05 | |
| RC | y= -0.7534x^2^+16.014x+263.27 , r^2^=0.9959 | | 5225.55 | 159.45 | |

WT: wild type; RC: root specific expression *OSCIPK2* transgenic rice.

Table S5 Grain yields and its components from root-specific overexpressed *OSCIPK2* (RC) rice and wild type (WT) rice in pot experiment under the low nitrogen condition.

| Genotype | Effective panicles (pot^-1^) | Spikelets per panicle | Seed setting rate (%) | 1000-grain weight (g) | Grain yield (g. pot^-1^) |
| --- | --- | --- | --- | --- | --- |
| RC | 17.01a | 46.10a | 92.84a | 24.88a | 24.08a |
| WT | 12.00b | 44.37b | 90.53b | 24.87a | 15.98b |

Different small letters in the same column represents significant difference (*p* < 0.05) calculated by LSD’s test.

Table S6 The ID of nitrogen-fixing bacteria strains in rice root.

| Species | %identity ^a^ | ID(ACCC)^b^ | Stock^c^ | **Phylum** | **Class** | **Order** | **Family** | **Genus** |
| --- | --- | --- | --- | --- | --- | --- | --- | --- |
| *Phenylobacterium* sp. | 100 | ACCC61373 | R2081 | Proteobacteria | Alphaproteobacteria | Caulobacterales | Caulobacteraceae | Phenylobacterium |
| *Rhizobium* sp. | 99.47 | ACCC60959 | R1241 | Proteobacteria | Alphaproteobacteria | Rhizobiales | Rhizobiaceae | Rhizobium |
| *Sphingomonas* sp. | 98.94 | ACCC60824 | R851 | Proteobacteria | Alphaproteobacteria | Sphingomonadales | Sphingomonadaceae | Sphingomonas |
| *Pleomorphomonas* sp. | 98.93 | ACCC61076 | R1405 | Proteobacteria | Alphaproteobacteria | Rhizobiales | Methylocystaceae | Pleomorphomonas |
| *Devosia* sp. | 98.14 | ACCC61424 | R2168 | Proteobacteria | Alphaproteobacteria | Rhizobiales | Hyphomicrobiaceae | Devosia |
| *Azospirillum* sp. | 97.11 | ACCC60862 | R919 | Proteobacteria | Alphaproteobacteria | Rhodospirillales | Rhodospirillaceae | Azospirillum |

(a) Sequence identity coverage; (b) The ID of ACCC (Agricultural Culture Collection of China, https://www.iarrp.cn/en/aboutus/structure/scientific/287900.htm); (c) The ID of rice root bacterial culture collection reported by zhang *et al.*(2019)

Table S7 Screening results of differential expressed root metabolites in root-specific overexpressed *OSCIPK2* (RC) rice compared to wild type (WT) rice

| Index | CAS | Compounds | WT | RC | VIP | Fold_Change | Type |
| --- | --- | --- | --- | --- | --- | --- | --- |
| **Flavonoids** | | | | | | | |
| mws0043 | 478-01-3 | Nobiletin | 2.20E+04 | 7.34E+04 | 2.01 | 3.33 | up |
| mws1661 | 520-27-4 | Diosmin | 1.21E+04 | 2.39E+05 | 1.43 | 19.7 | up |
| pmb0624 | - | 6-C-Hexosyl-luteolin O-hexoside | 8.13E+03 | 1.81E+04 | 1.14 | 2.22 | up |
| pmb0736 | - | Tricin 7-O-hexoside | 2.81E+05 | 5.92E+05 | 1.68 | 2.11 | up |
| pmb3041 | - | Tricin O-saccharic acid | 3.46E+05 | 8.68E+05 | 1.21 | 2.51 | up |
| pmb3042 | - | Tricin 5-O-hexoside | 2.93E+05 | 1.17E+06 | 1.67 | 3.99 | up |
| pme0088 | 491-70-3 | Luteolin | 9.37E+02 | 3.92E+03 | 1.84 | 4.18 | up |
| pme0368 | 552-57-8 | Apigenin 7-rutinoside(Isorhoifolin) | 3.67E+03 | 6.56E+04 | 1.65 | 17.86 | up |
| pme1605 | 17297-56-2 | Kaempferol 3-O-robinobioside(Biorobin) | 5.03E+03 | 7.67E+04 | 1.21 | 15.25 | up |
| pmp000194 | - | Malonyglygenistin | 9.00E+00 | 4.14E+04 | 1.5 | 4596.63 | up |
| pmp000579 | - | Diosmetin-7-O-galactoside | 8.67E+03 | 3.91E+04 | 1.57 | 4.51 | up |
| pmp000595 | 257-724-7 | Luteolin-7,3'-Di-O-β-D-Glucoside | 8.80E+04 | 1.83E+05 | 1.37 | 2.08 | up |
| mws0048 | 3681-93-4 | Vitexin | 9.50E+04 | 2.71E+04 | 1.94 | 0.28 | down |
| mws0566 | 452-86-8 | 4-Methylcatechol | 9.53E+02 | 3.55E+02 | 1.19 | 0.37 | down |
| mws0914 | 548-82-3 | Pinobanksin | 3.29E+05 | 6.34E+04 | 1.16 | 0.19 | down |
| mws1140 | 73692-50-9 | Naringenin chalcone(4,2',4',6'-Tetrahydroxychalcone) | 3.83E+05 | 7.30E+04 | 1.16 | 0.19 | down |
| mws1434 | 29702-25-8 | Isovitexin | 1.05E+05 | 2.48E+04 | 1.91 | 0.24 | down |
| pme0376 | 480-41-1 | Naringenin | 3.44E+05 | 6.51E+04 | 1.2 | 0.19 | down |
| pmp000413 | 66026-80-0 | Genistein 8-C-glucoside | 7.26E+04 | 1.93E+04 | 1.89 | 0.27 | down |
| pmp000571 | 520-36-5 | Apigenin | 6.98E+03 | 3.22E+03 | 1.43 | 0.46 | down |
| **Phenolic acids** | | | | | | | |
| mws0178 | 327-97-9 | Chlorogenic acid | 9.00E+00 | 4.64E+04 | 1.39 | 5152.19 | up |
| mws0906 | 531-29-3 | Coniferin | 9.00E+00 | 5.45E+05 | 2.06 | 60555.56 | up |
| pme0085 | 537-15-5 | Rosmarinic acid | 1.17E+04 | 4.27E+04 | 1.65 | 3.65 | up |
| pmf0424 | 14534-61-3 | Isochlorogenic acid B | 2.75E+03 | 5.61E+03 | 2.01 | 2.04 | up |
| pmn001420 | - | 1-O-[(E)-Caffeoyl]-β-D-glucopyranose | 9.00E+00 | 5.95E+05 | 2.06 | 66074.07 | up |
| **Organic acids** | | | | | | | |
| mws0208 | 124-04-9 | Adipic Acid | 4.69E+04 | 1.18E+05 | 1.74 | 2.51 | up |
| mws0281 | 77-92-9 | Citric Acid | 2.74E+06 | 8.33E+06 | 1.48 | 3.04 | up |
| mws0425 | 498-23-7 | Citraconic acid | 9.87E+04 | 2.13E+05 | 1.38 | 2.16 | up |
| pmb2867 | - | Kynurenic acid O-hexside | 1.32E+03 | 4.20E+03 | 1.23 | 3.19 | up |
| Terpenoids | | | | | | | |
| pmn001709 | - | Asiatic acid(isomers) | 4.25E+03 | 9.27E+03 | 1.86 | 2.18 | up |
| mws1610 | 4373-41-5 | Maslinic acid | 1.89E+05 | 6.41E+04 | 1.23 | 0.34 | down |
| pmn001706 | 26707-60-8 | 2-Hydroxyoleanolic acid | 3.03E+05 | 8.39E+04 | 1.51 | 0.28 | down |
| Alkaloids | | | | | | | |
| mws0352 | 58-55-9 | Theophylline | 9.00E+00 | 2.25E+04 | 2.06 | 2500 | up |
| mws0191 | 107-43-7 | Betaine | 1.43E+06 | 7.23E+04 | 1.57 | 0.05 | down |
| Nucleotides and derivatives | | | | | | | |
| pmb0981 | 61-19-8 | Adenosine 5'-monophosphate | 3.74E+04 | 7.52E+04 | 1.24 | 2.01 | up |
| Amino acids and derivatives | | | | | | | |
| mws0219 | 56-84-8 | L-AsparticAcid | 1.15E+04 | 3.16E+04 | 1.38 | 2.75 | up |
| Lignans and Coumarins | | | | | | | |
| pmn001501 | 31008-19-2 | Fargesin | 8.72E+03 | 3.12E+04 | 1.57 | 3.58 | up |
| Others | | | | | | | |
| pmn001399 | - | 2,4,6,4'-Tetrahydroxy-stilbene-2-O-D-glucopyranoside | 29000 | 28200 | 1.93 | 2.08 | up |

WT, wild type and RC, *OSCIPK2*-overexpressing transgenic rice plant with root specific promoter (RC).

**Table S8 Screening results of differential expressed soil metabolites in root-specific overexpressed *OSCIPK2* (RC) rhizosphere compared to wild type (WT) rhizosphere**

| Compounds | CAS | RT | WT | RC | VIP | p_value | Fold_Change | type |
| --- | --- | --- | --- | --- | --- | --- | --- | --- |
| **Organic acid** |  |  |  |  |  |  |  |  |
| Oxalic acid | 144-62-7 | 23.281 | 0.012599671 | 0.006228245 | 1.39 | 8.37E-09 | 0.57 | Down |
| Fumaric acid | 110-17-8 | 36.05 | 0.007796935 | 0.011823634 | 1.38 | 1.49E-08 | 1.74 | Up |
| Malic acid | 6915-15-7 | 18.988 | 0.006688099 | 0.010046165 | 1.40 | 1.02E-10 | 1.72 | Up |
| Citric acid | 77-92-9 | 24.204 | 0.01001939 | 0.014781806 | 1.28 | 7.01E-05 | 1.69 | up |
| Succinic acid | 110-15-6 | 19.764 | 0.011905808 | 0.017299005 | 1.38 | 7.68E-08 | 1.66 | Up |
| Lignoceric acid | 557-59-5 | 28.718 | 0.011021849 | 0.016408332 | 1.38 | 2.55E-08 | 1.70 | Up |
| Palmitic Acid | 57-10-3 | 21.998 | 0.021209021 | 0.029767661 | 1.39 | 7.33E-09 | 1.61 | Up |
| **Alcohol** |  |  |  |  |  |  |  |  |
| Propanediol | 57-55-6 | 24.755 | 0.001373625 | 0.000750109 | 1.38 | 9.51E-09 | 0.63 | Down |
| Phenylethyl Alcohol | 19601-20-8 | 20.48 | 0.027109302 | 0.0033714 | 1.41 | 1.54E-12 | 0.14 | Down |
| Ethylene glycol | 107-21-1 | 5.859 | 0.006648135 | 0.01206985 | 1.39 | 4.26E-09 | 2.08 | Up |
| Xylitol | 87-99-0 | 16.314 | 0.003145321 | 0.005699738 | 1.40 | 4.1E-10 | 2.08 | Up |
| 1-deoxyerythritol | 3068-00-6 | 21.43 | 0.006864835 | 0.00269651 | 1.39 | 3.37E-09 | 0.45 | Down |
| **Sugar** |  |  |  |  |  |  |  |  |
| D-Glucose | 9050-36-3 | 20.48 | 0.009075684 | 0.043412132 | 1.41 | 1.17E-12 | 5.48 | Up |
| Sucrose | 57-50-1 | 27.172 | 0.00262825 | 0.012205548 | 1.41 | 2.62E-12 | 5.32 | Up |
| d-Mannose | 3458-28-4 | 16.684 | 0.017516793 | 0.031703244 | 1.40 | 3.27E-09 | 2.07 | Up |
| **Terpenes** |  |  |  |  |  |  |  |  |
| Stigmasterol | 83-48-7 | 32.719 | 0.006947449 | 0.011222262 | 1.39 | 4.34E-08 | 1.85 | Up |
| Campesterol | 84-74-2 | 32.434 | 0.00265016 | 0.003493525 | 1.38 | 6.35E-08 | 1.51 | Up |
| **Ester** |  |  |  |  |  |  |  |  |
| α-ketoglutarate | 144509-68-2 | 24.003 | 0.003199358 | 0.004504804 | 1.38 | 3.02E-08 | 1.61 | Up |
| Dibutyl phthalate | 84-74-2 | 20.589 | 0.011578231 | 0.006376263 | 1.38 | 3.79E-08 | 0.63 | Down |
| **Amino acids** |  |  |  |  |  |  |  |  |
| L-Aspartate | 7675-83-4 | 21.285 | 0.003441962 | 0.0056137 | 1.39 | 1.94E-09 | 1.87 | Up |
| glycine | 56-40-6 | 21.482 | 0.003369531 | 0.005524608 | 1.39 | 8.53E-10 | 1.88 | Up |
| **Others** |  |  |  |  |  |  |  |  |
| gluconic acid lactone | 90-80-2 | 14.694 | 0.002256235 | 0.004191446 | 1.39 | 1.05E-09 | 2.13 | Up |
| Acetamide | 60-35-5 | 11.605 | 0.007348101 | 0.018231557 | 1.40 | 2.68E-09 | 2.84 | Up |

WT, wild type and RC, *OSCIPK2*-overexpressing transgenic rice plant with root specific promoter (RC).

**Text S1、Mass spectrometer parameters of LC-MS.**

**UPLC Conditions:**

The analytical conditions were as follows, UPLC: column, Waters ACQUITY UPLC HSS T3 C18 (1.8 μm, 2.1 mm*100 mm); The mobile phase was consisted of solvent A, pure water with 0.04% acetic acid, and solvent B, acetonitrile with 0.04% acetic acid. Sample measurements were performed with a gradient program that employed the starting conditions of 95% A, 5 % B. Within 10 min, a linear gradient to 5% A, 95% B was programmed, and a composition of 5% A, 95% B was kept for 1min. Subsequently, a composition of 95% A,5.0 % B was adjusted within 0.10 min and kept for 2.9 min. The column oven was set to 40°C; The injection volume was 4μl. The effluent was alternatively connected to an ESI-triple quadrupole-linear ion trap (QTRAP)-MS.

**ESI-Q TRAP-MS/MS：**

LIT and triple quadrupole (QQQ) scans were acquired on a triple quadrupole-linear ion trap mass spectrometer (Q TRAP), API 4500 Q TRAP UPLC/MS/MS System, equipped with an ESI Turbo Ion-Spray interface, operating in positive and negative ion mode and controlled by Analyst 1.6.3 software (AB Sciex). The ESI source operation parameters were as follows: ion source, turbo spray; source temperature 550℃; ion spray voltage (IS) 5500 V (positive ion mode)/-4500 V (negative ion mode); ion source gas I (GSI), gas II(GSII), curtain gas (CUR) were set at 50, 60, and 30.0 psi, respectively; the collision gas(CAD) was high. Instrument tuning and mass calibration were performed with 10 and 100 μmol/L polypropylene glycol solutions in QQQ and LIT modes, respectively. QQQ scans were acquired as MRM experiments with collision gas (nitrogen) set to 5 psi. DP and CE for individual MRM transitions was done with further DP and CE optimization. A specific set of MRM transitions were monitored for each period according to the metabolites eluted within this period.

**Text S2: Mass spectrometer parameters of GC-MS.**

The soil metabolome were analyzed by gas chromatography-mass spectrometry (GC-MS) using a Shimadzu GC-2010 plus equipped with a Shimadzu TQ8040 triple-quadrupole MS (Shimadzu, Kyoto, Japan). The carrier gas He (99.999%) was flowed into 30 m × 0.25 mm ID × 0.25 μm df capillary column (SH-Rxi-5Sil MS, Shimadzu, Japan) by splitless mode, the column oven temperature procedure was as follow: 60 °C–250 °C by 8.5 °C /min, hold for 5 min, then 250 °C–300 °C by 15 °C /min, hold for 4.5 min. The samples were vaporized at 280 °C by injection port. The flow linear velocity was 42.8 cm/s, flow rate was set at 1.38 ml/min. Temperature of Ion source (EI) and interface were 200 and 280 °C respectively, the solvent cut time was 3 min, the ionization energy was 70 eV.
